# Supplementary material for: Single-crystal 2D covalent organic frameworks for high-capacity methane storage
Source: Nat Commun. 2026 Feb 14;17:2740. doi: 10.1038/s41467-026-69614-7 (PMC13013593; doi:10.1038/s41467-026-69614-7)
Supplement: Supplementary file 1 — Supplementary Information [file 41467_2026_69614_MOESM1_ESM.pdf]

Supplementary information

Single-crystal 2D Covalent Organic Frameworks for High-capacity Methane Storage

Baoqiu Yu[1,2], Felipe L. Oliveira[3,4], Wenliang Li[5], Qingmei Xu[2], Xu Ding[1], Shangwei Yuan[1], Yucheng Jin[2], Hua Liu[1], Hailong Wang[2✉], Xin Xiao[1✉], Jingping Zhang[5], Guillaume Maurin[3,4], Banglin Chen[6,7✉], and Jianzhuang Jiang[1,2✉]

[1] Guizhou Key Laboratory of Macrocyclic and Supramolecular Chemistry, School of Chemistry and Chemical Engineering, Guizhou University, Guiyang 550025, China.

[2] Beijing Key Laboratory for Science and Application of Functional Molecular and Crystalline Materials, Department of Chemistry and Chemical Engineering, School of Chemistry and Biological Engineering, University of Science and Technology Beijing, Beijing 100083, China.

[3] ICGM, Université de Montpellier, CNRS, ENSCM, Montpellier 34293, France.

[4] Institute Universitaire de France (IUF), Paris, France.

[5] Faculty of Chemistry, Northeast Normal University, Changchun 130024, China.

[6] Fujian Provincial Key Laboratory of Polymer Materials College of Chemistry & Materials Science, Fujian Normal University, Fuzhou 350007, China.

[7] Key Laboratory of the Ministry of Education for Advanced Catalysis Materials, College of Chemistry and Materials Science, Zhejiang Normal University, Jinhua 321004, China.

✉ Corresponding author. Email: hlwang@ustb.edu.cn (H.W.); xxiao@gzu.edu.cn (X.X.); banglin.chen@fjnu.edu.cn (B.C.); jianzhuang@ustb.edu.cn (J.J.).

Materials and Methods

General information. The 1,3,5-trimethoxy-2,4,6-tris(4-aminophenyl)benzene (TOAB) and 1,3,5-trimethoxy-2,4,6-tris(4-formylphenyl)benzene (TOFB) were prepared and optimized according to the published procedure.[1] 1,3,5-Trimethyl-2,4,6-tris(4-aminophenyl)benzene (TTAB, ≥ 95%), and 1,3,5-trimethyl-2,4,6-tris(4-formylphenyl)benzene (TTFB, ≥ 95%) were purchased from Jilin Chinese Academy of Sciences-Yanshen Technology Co., Ltd. Aniline (GC, ≥ 99.98%) was purchased from Shanghai Aladdin Biochemical Technology Co., Ltd. Super dry 1,4-dioxane (AR, ≥ 99.7%,) was purchased from Meryer (Shanghai) Chemical Technology Co., Ltd. Super dry mesitylene (AR, ≥ 99%,) was purchased from Sinopharm Chemical Reagent Beijing Co. Ltd. Trichloromethane (TCM) was purchased from Sinopharm Group Chemical Reagent Beijing Co., Ltd. The other chemicals were obtained from commercial sources and used without further purification.

Physical characterization. NMR spectra were recorded on a Bruker DPX 400 spectrometer (^{1}H: 400 MHz, ^{13}C: 100 MHz). ^{1}H NMR spectra were referenced internally using the residual solvent resonances (δ = 2.50 ppm for DMSO-d_6) relative to $SiMe_4$. ^{13}C NMR spectrum was referenced internally by using the solvent resonance (δ = 39.50 ppm for DMSO-d_6). Powder X-ray diffraction (PXRD) was collected at room temperature on a PANalytical Empyrean series 3 diffractometer equipped with Cu Kα radiation operating at 45 kV and 40 mA and on a diffracted-beam graphite monochromator. Fourier transform infrared (FT-IR) spectra of COFs in a transmittance (%) mode was collected on pressed KBr pellets at room temperature. The gas sorption isotherms were measured on a Micromeritics ASAP 2020 PLUS HD88 surface area analyzer, and the measurement temperature was maintained at 77 K with a liquid nitrogen bath, 273 K with an ice-water bath, and 298 K with a water bath in an air-conditioned 25 °C laboratory. Before measurement, the samples were degassed in vacuum at 100 °C for 12 h. To estimate pore size distributions, density functional theory (DFT) was applied to analyze the N_2 isotherm based on the model of N_2-DFT Model with cylinder pores and the method of non-negative regularization. Scanning electron microscopy (SEM) image was recorded on a GeminiSEM 300 scanning electron microscope. Transmission electron microscopy (TEM) images were collected from HT7700 electron microscope at 100 KV. All the solid-state nuclear magnetic resonance (SSNMR) experiments were performed on activated phase with magic angle spinning (MAS) on a Bruker Avance II 400 MHz wide-bore solid-state NMR spectrometer at a magnetic field of 9.4 T.

Three-dimensional electron diffraction (3D ED). The 3D ED data of GZU-1, GZU-2, and GZU-3 were collected in ReadCrystal Biotechnology Co., Ltd in Suzhou by using a JEOL 2100-plus transmission electron microscope equipped with MerlinEM direct electron detector under 200 kV acceleration voltage. The COF powder was dusted onto a copper grid and transferred to the transmission electron microscope with a Fischone 2550 cryo-holder. The holder dewar was then filled with liquid nitrogen. The 3D ED datasets were collected after the holder tip was cooled to 77 K. During the data collection, the TEM goniometer was rotated continuously while the ED patterns were captured from the crystal simultaneous, and all the ED patterns were recorded under the spot size 4 with the exposure time 1.0 s. Data processing was conducted using the software package XDS[2] and REDp[3].

The solution of both single-crystal COFs was performed by SHELXT-2018[4] with the merged and scaled datasets. All the positions of non-hydrogen atoms could be directly located in the initial structural model. In the following up refinement process, we only use the Simulated Annealing method to disturb the rotation

direction of benzene ring to determine the most suitable atomic position. Structure refinement against the *c*RED data were performed by the SHELXL software package[5]. The intensity of crystal data for COFs collected by 3D ED is much lower than that collected by single crystal X-ray diffraction, therefore being not able to be amenable to absorption correction. As a result, the anisotropic refinement would result in a large temperature factor in one axial direction and thus the atomic distortion. As a result, all the carbon and nitrogen atoms of GZU-1, GZU-2, and GZU-3 have been refined isotropically, with soft restraints for the geometry of the phenyl ring and the C-C and C=N bond lengths. Hydrogen atoms with isotropic parameters were added geometrically and refined using a riding model. CCDC 2455725, 2415331, and 2415329 for GZU-1, GZU-2, and GZU-3 contains the supplementary crystallographic data for this paper. These data can be obtained free of charge from the Cambridge Crystallographic Data Centre *via* www.ccdc.cam.ac.uk/data_request/cif.

Supplementary Fig. 1. The synthesis route of TOFB.

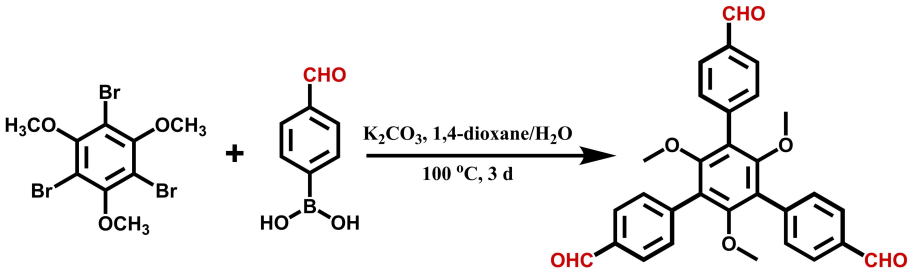

Synthesis of 1,3,5-trimethoxy-2,4,6-tris(4-formylphenyl)benzene (TOFB). To a 500 mL Schlenk flask charged with compound 1,3,5-tribromo-2,4,6-trimethoxybenzene (1.21 g, 3 mmol), 4-formylphenylboronic acid (2.25 g, 15 mmol), K_2CO_3 (14.8 g, 45 mmol) and $Pd(PPh_3)_4$ (0.115 g, 0.1 mmol) was added 100 mL degassed 1,4-dioxane and 20 mL degassed H_2O. The mixture was further degassed by four freeze-pump-thaw cycles. Subsequently, the reaction mixture was heated to 100 °C and kept for 3 days under a N_2 atmosphere. After cooling to room temperature, the solvent was evaporated under reduced pressure. The resulting mixture was poured into water and extracted with dichloromethane (3 × 100 mL). The combined organic layers were dried over anhydrous $MgSO_4$ and filtered. The organic solvent was removed by evaporation under reduced pressure and the crude product was purified by alumina chromatography column with ethyl acetate/petroleum ether (1:1 v/v) as eluent to obtained compound TOFB as a white solid (1.26 g, 88 %). ^1H NMR (DMSO-d_6, 400 MHz): δ (ppm) 2.98 (s, 9H), 7.71 (d, 6H), 7.99 (d, 6H), 10.06 (s, 3H). ^{13}C NMR (DMSO-d_6, 100 MHz): δ (ppm) 60.55, 124.88, 129.08, 131.18, 135.05, 139.94, 155.87, and 192.86.

Supplementary Fig. 2. The synthesis route of TOAB.

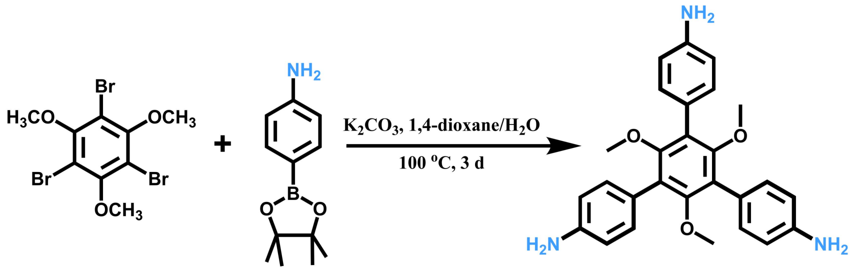

Synthesis of 1,3,5-trimethoxy-2,4,6-tris(4-aminophenyl)benzene (TOAB). To a 500 mL Schlenk flask charged with compound 1,3,5-tribromo-2,4,6-trimethoxybenzene (1.21 g, 3 mmol), 4-aminophenylboronic

acid pinacol ester (3.29 g, 15 mmol), K_2CO_3 (14.8 g, 45 mmol) and $Pd(PPh_3)_4$ (0.115 g, 0.1 mmol) was added 100 mL degassed 1,4-dioxane and 20 mL degassed H_2O. The mixture was further degassed by four freeze-pump-thaw cycles. Subsequently, the reaction mixture was heated to 100 °C and kept for 3 days under a N_2 atmosphere. After cooling to room temperature, the solvent was evaporated under reduced pressure. The resulting mixture was poured into water and extracted with dichloromethane (3 × 100 mL). The combined organic layers were dried over anhydrous $MgSO_4$ and filtered. The organic solvent was removed by evaporation under reduced pressure and the crude product was purified by alumina chromatography column with ethyl acetate/petroleum ether (1:1 v/v) as eluent to obtained compound TOAB as a white solid (0.95 g, 72 %). ^{1}H NMR (DMSO-d_6, 400 MHz): δ (ppm) 3.09 (s, 9H), 3.69 (s, 6H), 6.73 (d, 6H), 7.29 (d, 6H). ^{13}C NMR (DMSO-d_6, 100 MHz): δ (ppm) 59.53, 113.42, 121.43, 125.74, 130.81, 147.32, and 155.06.

Formation energy comparison. To gain deeper insight into the factors responsible for the superior crystallinity observed in the GZU COFs, DFT calculations were carried out to investigate their relative thermodynamic stability. These results were compared with those of other well-known COFs previously reported in the literature, including DISTAP-1[6], TPB-DMTP[7], COF-701[8], TTI-COF[9], and HHTP-DPB[10]. The calculated formation energies, summarized in Fig. 4a), reveal a clear stability trend among the GZU materials. Specifically, GZU-2 displays the most favorable formation energy at −172 kJ·mol^{-1} per sheet, followed by GZU-3 (−167 kJ·mol^{-1} per sheet) and GZU-1 (−165 kJ·mol^{-1} per sheet).

Notably, these values are significantly more exergonic - that is, more negative - than those obtained for the reference COFs, which exhibit formation energies of −113, −106, −103, −71, and −59 kJ·mol^{-1} per sheet for TPB-DMTP, DISTAP-1, COF-701, TTI-COF, and HHTP-DPB, respectively. This substantial difference highlights the high thermodynamic stability of the GZU series compared to other structurally related frameworks.

II. Characterization and properties.

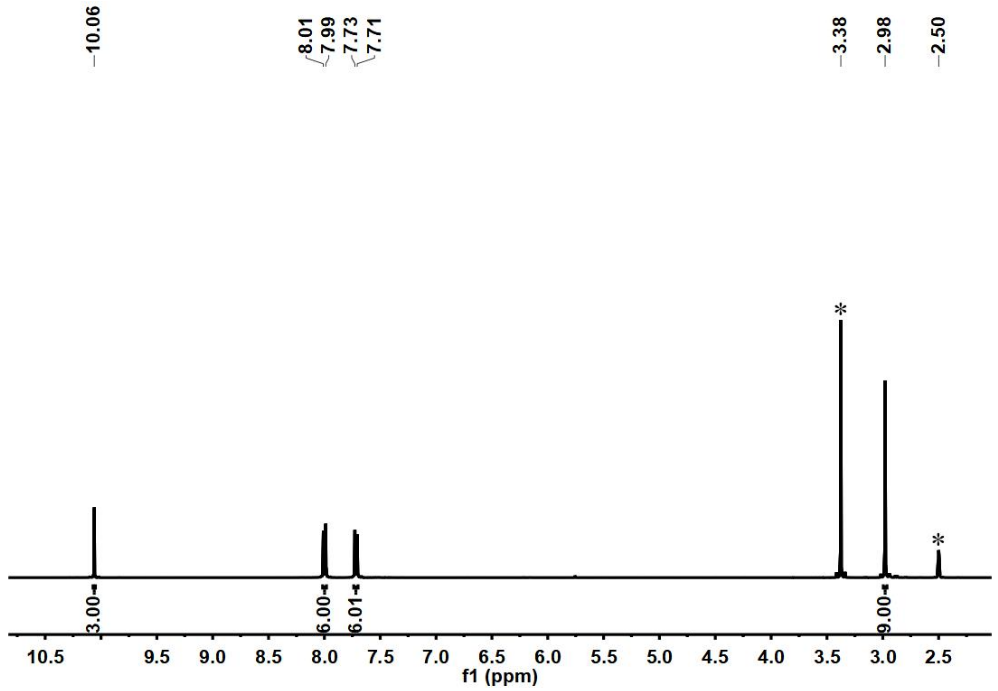

Supplementary Fig. 3. The ^{1}H NMR spectrum (400 MHz) of TOFB in DMSO-d_6. *denotes the solvent impurity in DMSO-d_6.

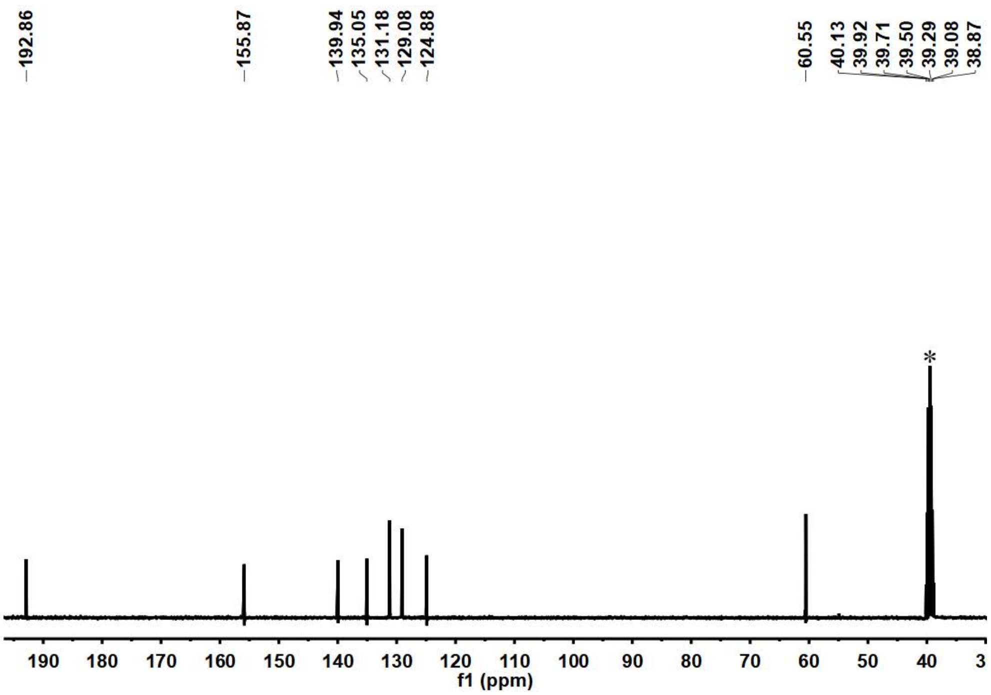

Supplementary Fig. 4. The ^{13}C NMR spectrum (100 MHz) of TOFB in DMSO-d_6. *denotes the solvent impurity in DMSO-d_6.

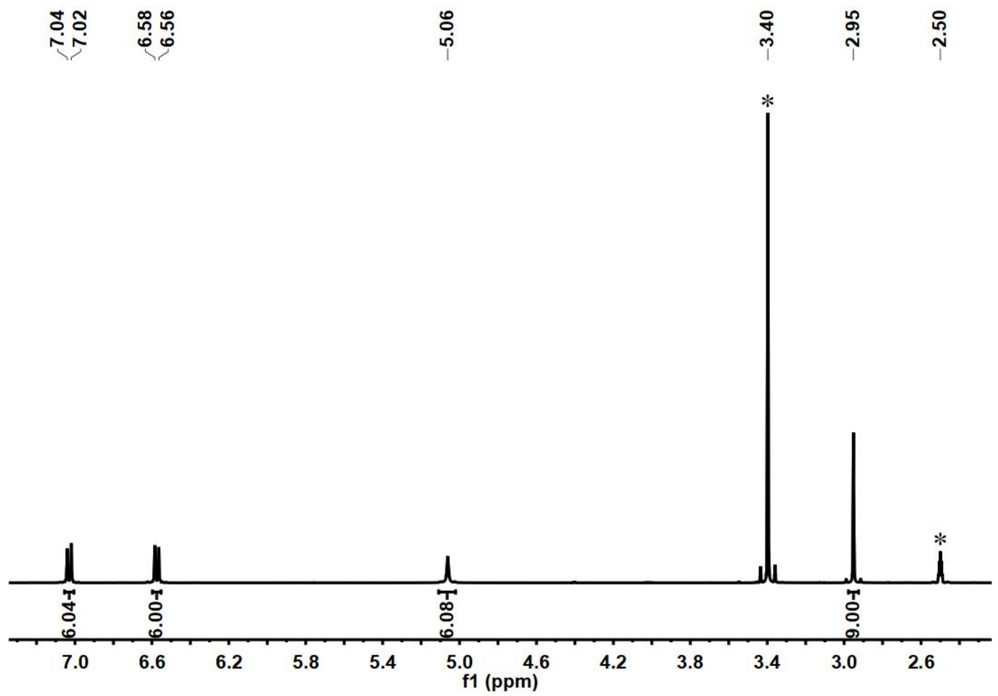

Supplementary Fig. 5. The ^1H NMR spectrum (400 MHz) of TOAB in DMSO-d_6. *denotes the solvent impurity in DMSO-d_6.

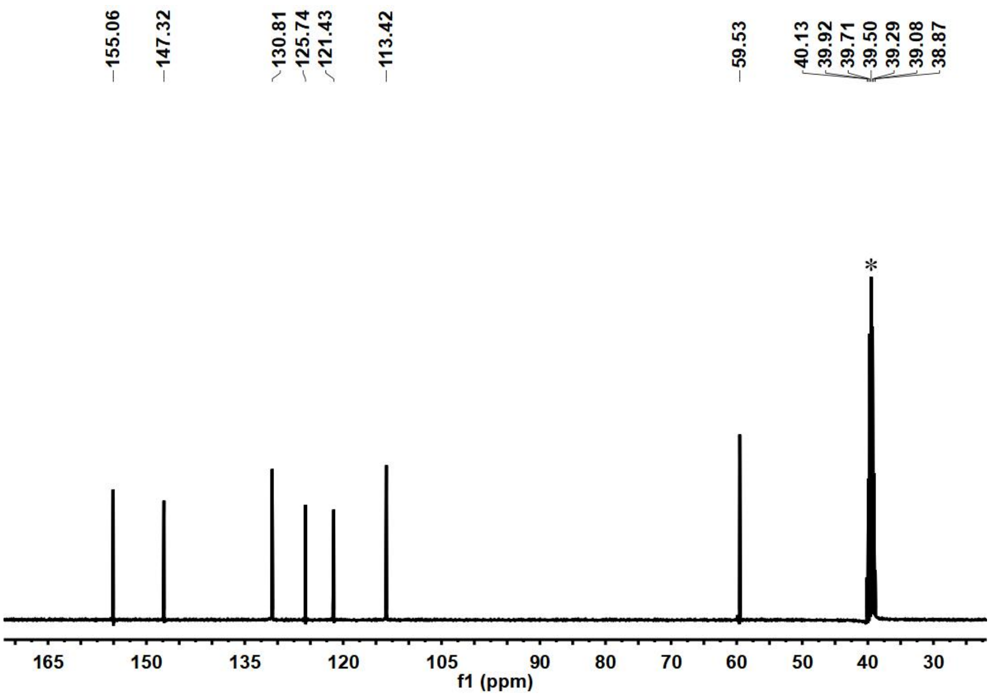

Supplementary Fig. 6. The ^{13}C NMR spectrum (100 MHz) of TOAB in DMSO-d_6. *denotes the solvent impurity in DMSO-d_6.

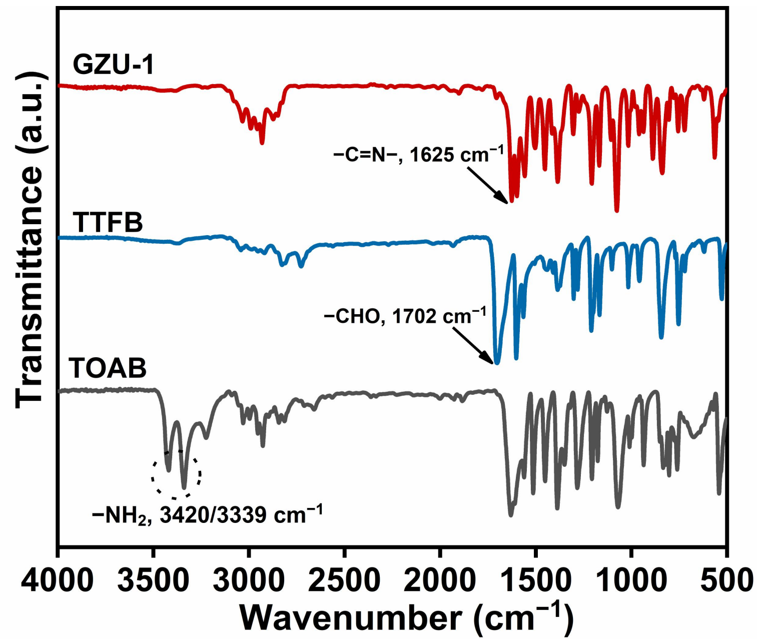

Supplementary Fig. 7. The FT-IR spectra of activated GZU-1 in comparison with that of TTFB and TOAB.

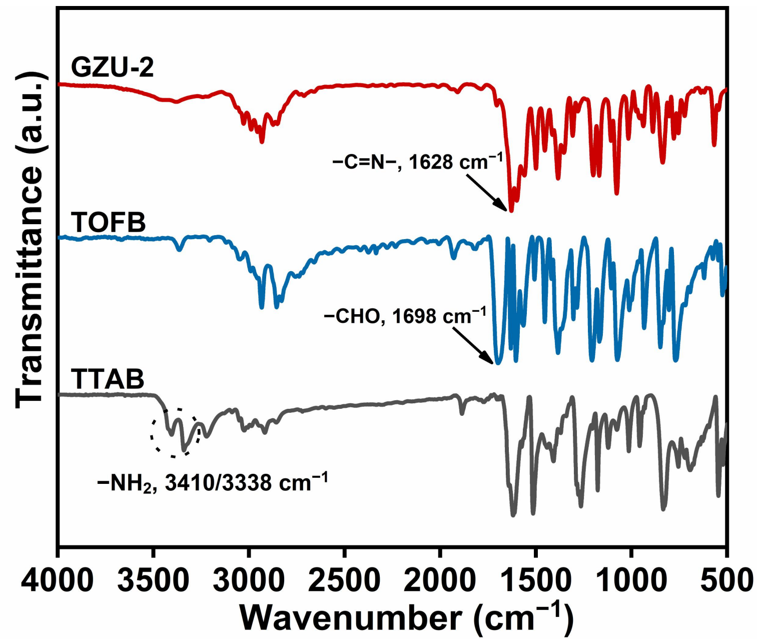

Supplementary Fig. 8. The FT-IR spectra of GZU-2 in comparison with that of TOFB and TTAB.

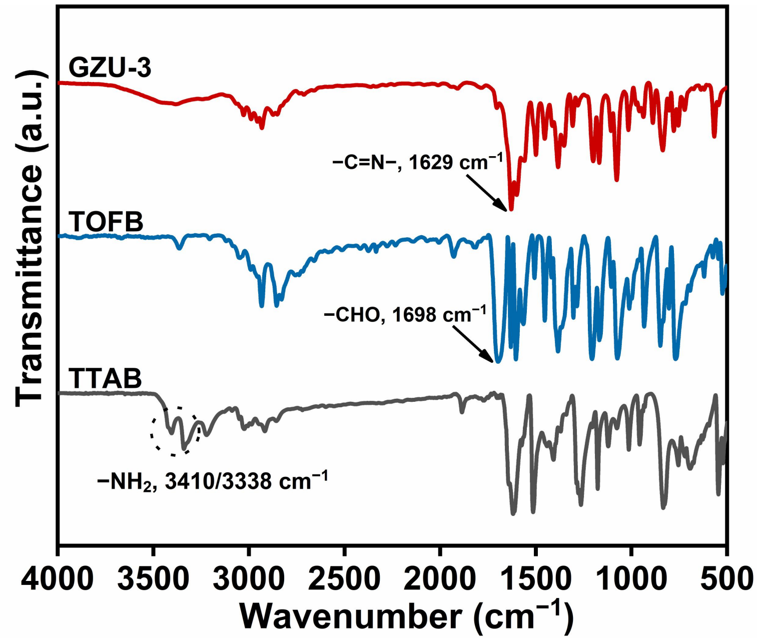

Supplementary Fig. 9. The FT-IR spectra of GZU-3 in comparison with that of TOFB and TTAB.

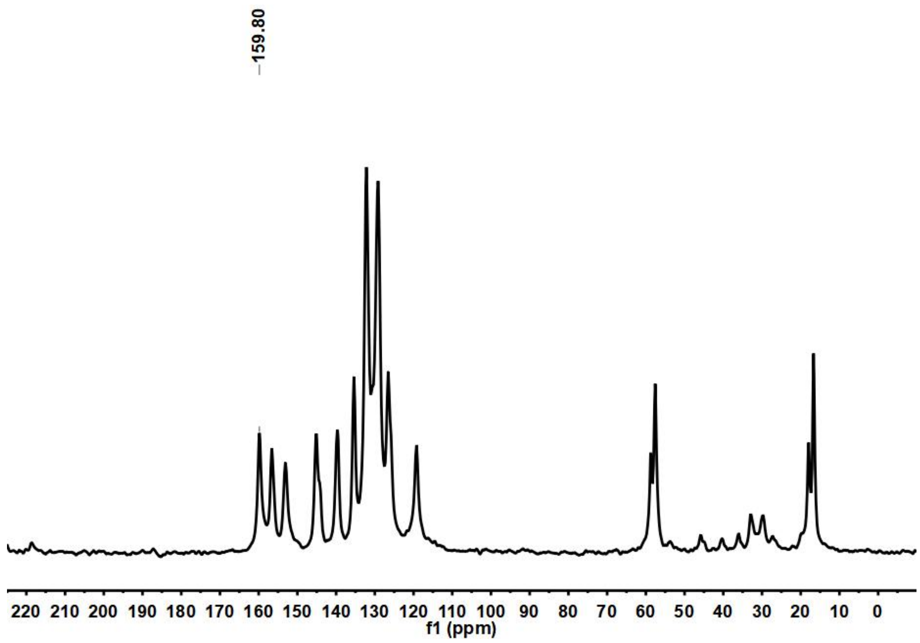

Supplementary Fig. 10. The ^{13}C CP/MAS spectrum of GZU-1.

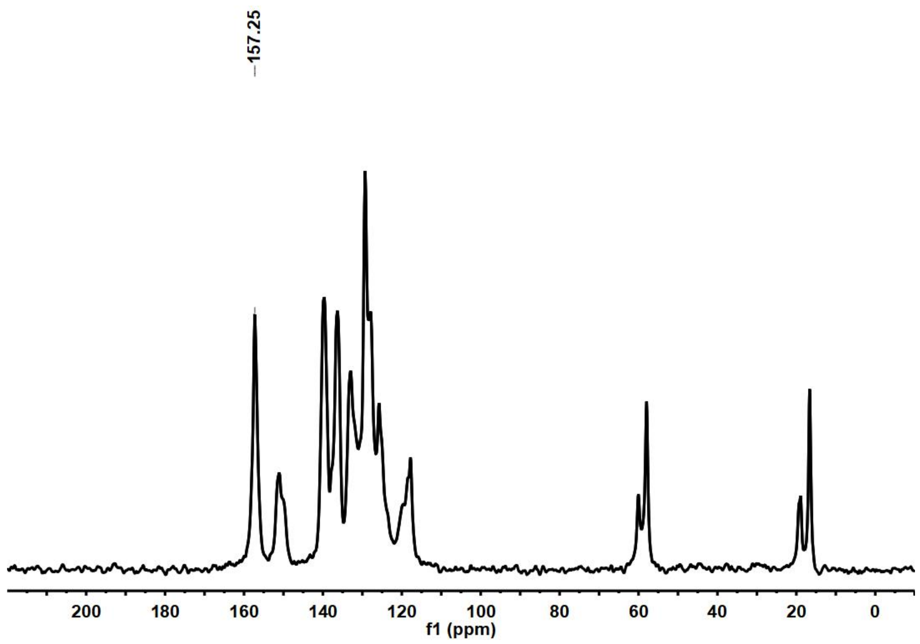

Supplementary Fig. 11. The ^{13}C CP/MAS spectrum of GZU-2.

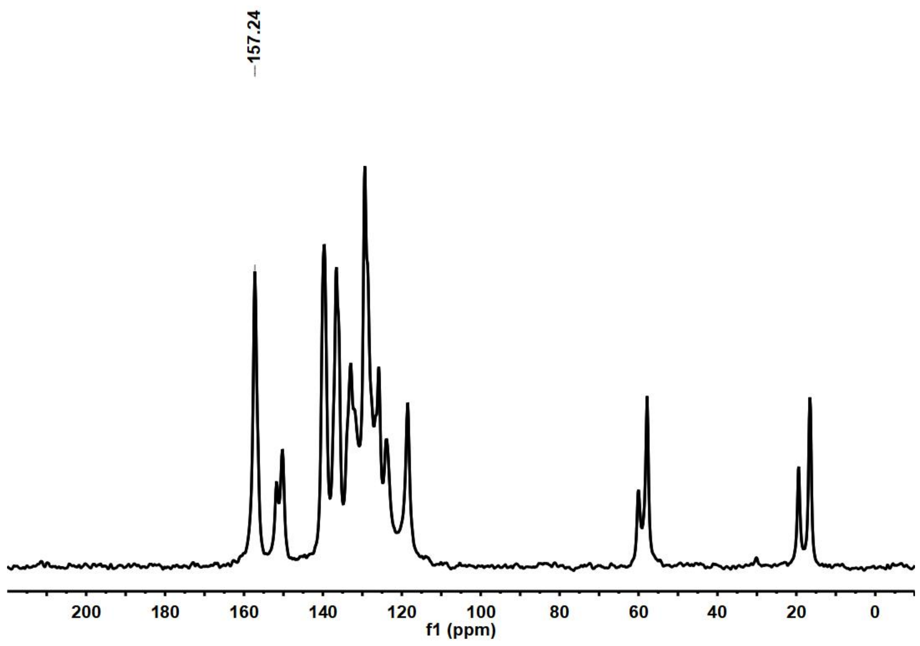

Supplementary Fig. 12. The ^{13}C CP/MAS spectrum of GZU-3.

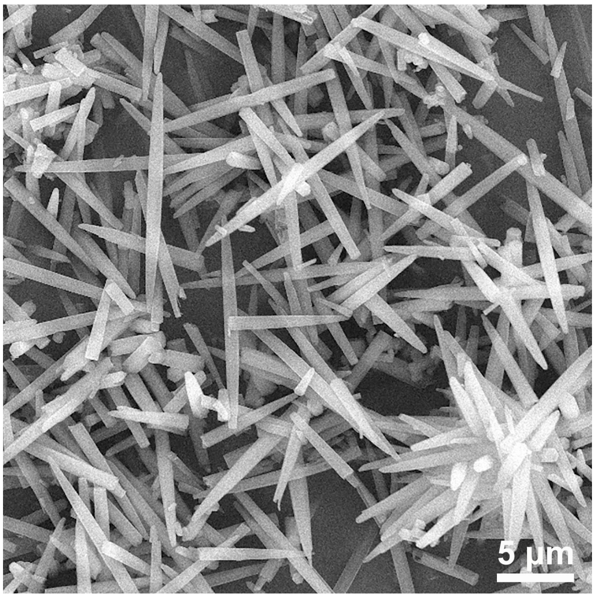

Supplementary Fig. 13. The SEM image of single-crystal GZU-1.

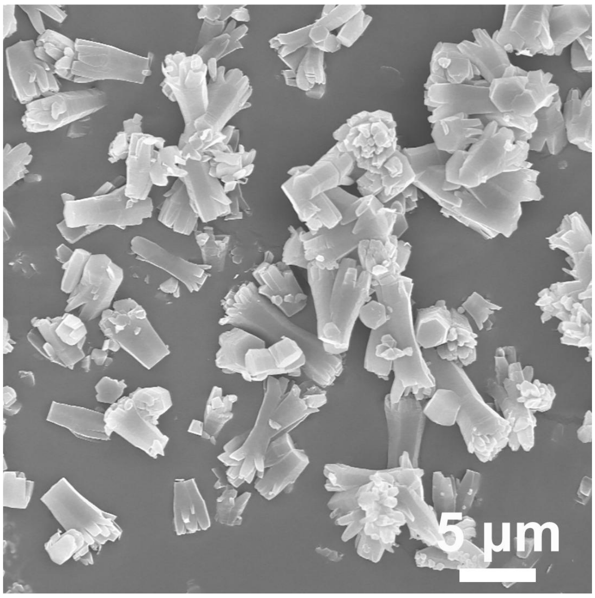

Supplementary Fig. 14. The SEM image of single-crystal GZU-2.

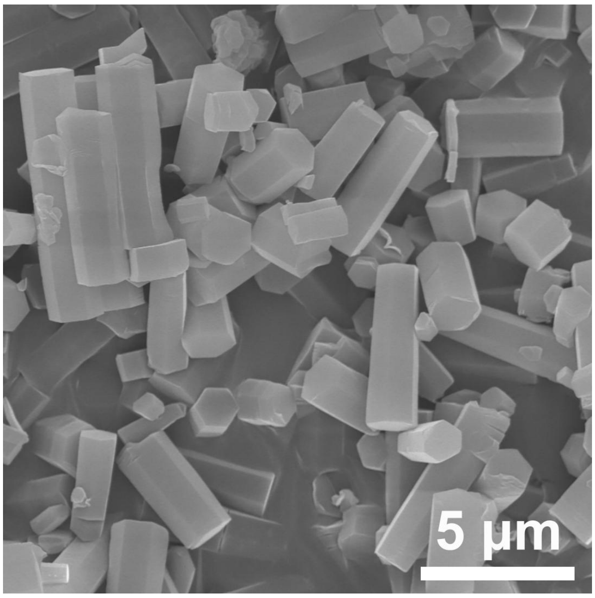

Supplementary Fig. 15. The SEM image of single-crystal GZU-3.

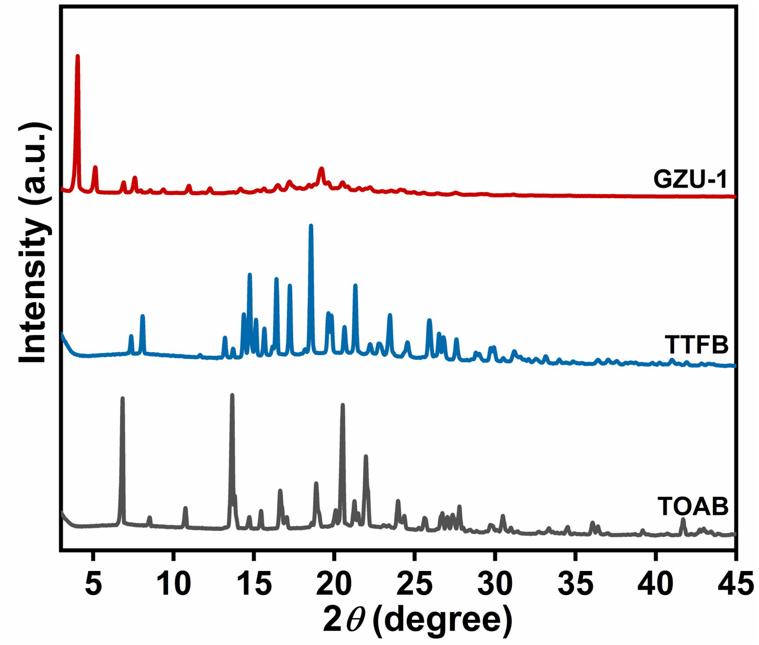

Supplementary Fig. 16. The PXRD patterns of single-crystal GZU-1, TTFB, and TOAB.

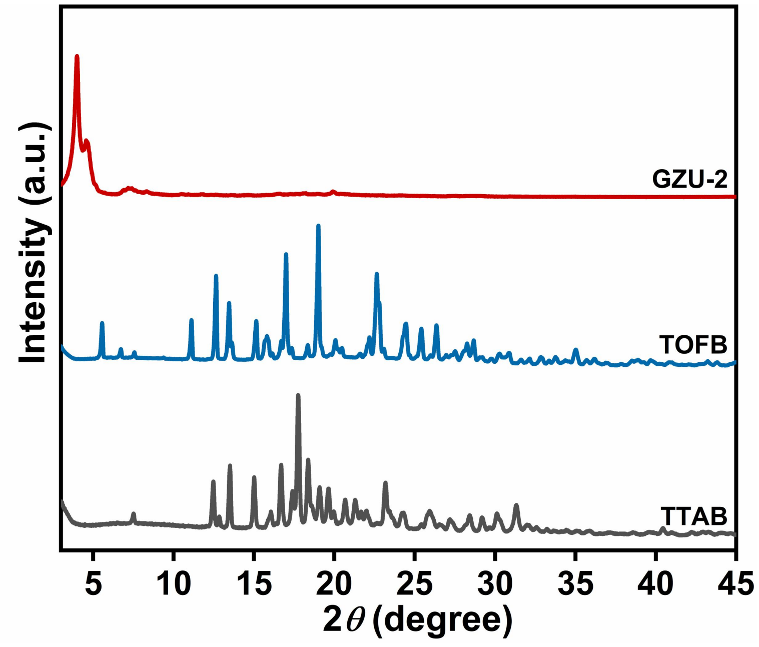

Supplementary Fig. 17. The PXRD patterns of single-crystal GZU-2, TOFB, and TTAB.

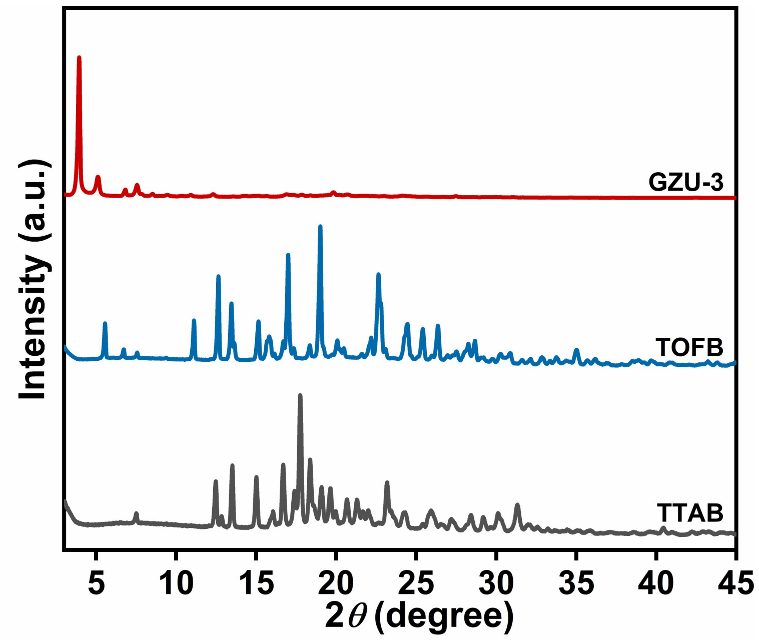

Supplementary Fig. 18. The PXRD patterns of single-crystal GZU-3, TOFB, and TTAB.

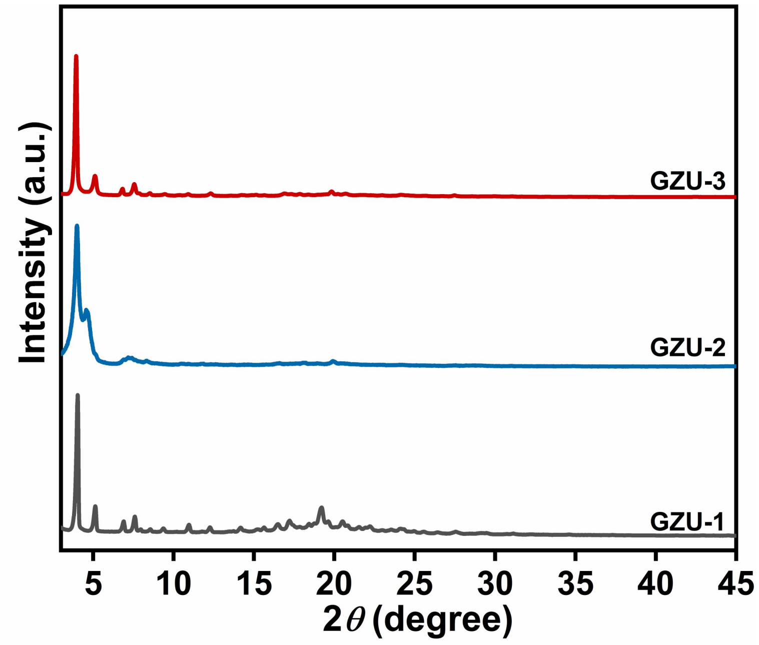

Supplementary Fig. 19. The PXRD patterns of single-crystal GZU-1, GZU-2, and GZU-3.

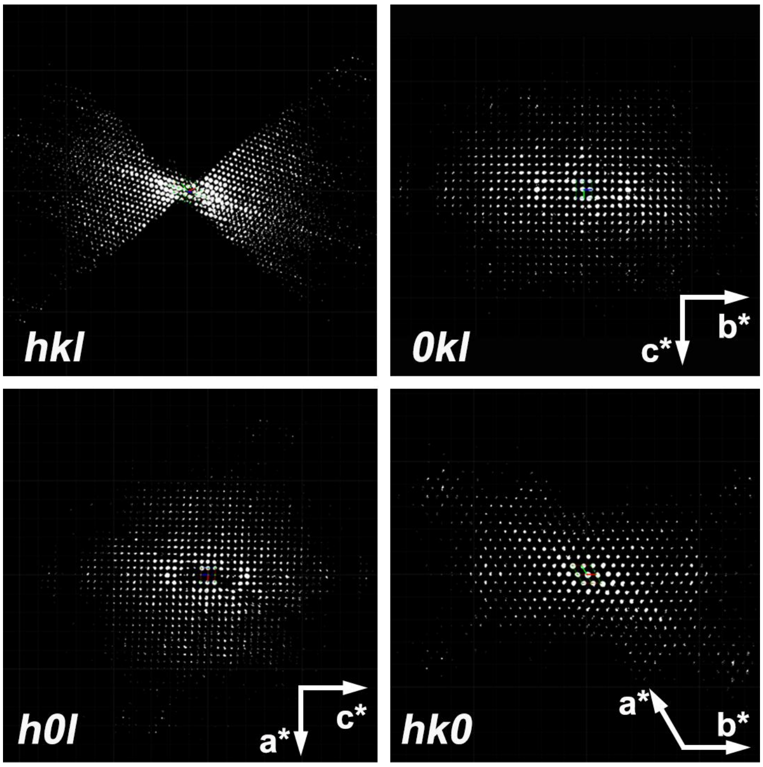

Supplementary Fig. 20. The projections of the 3D ED data of single-crystal GZU-1.

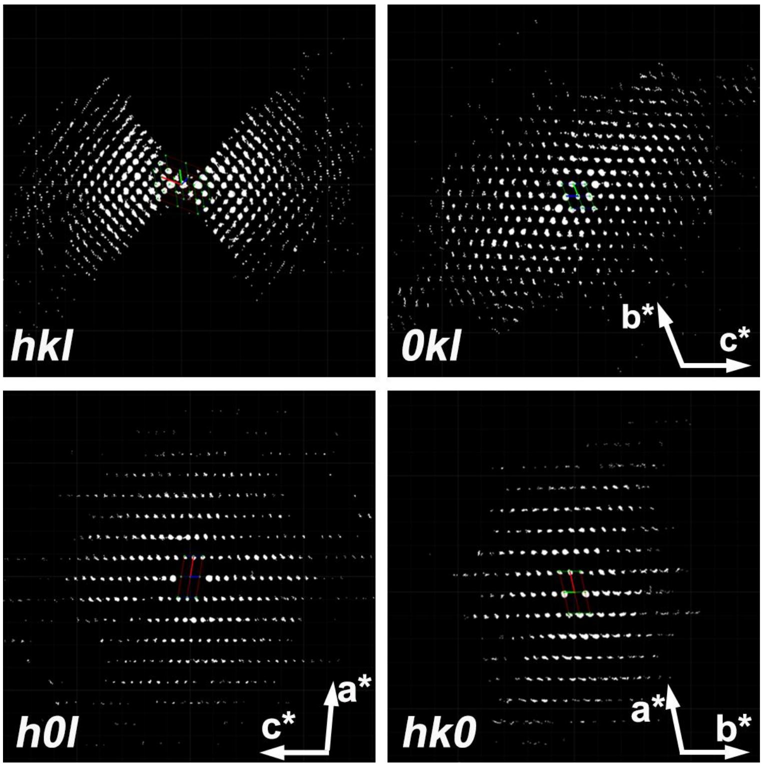

Supplementary Fig. 21. The projections of the 3D ED data of single-crystal GZU-2.

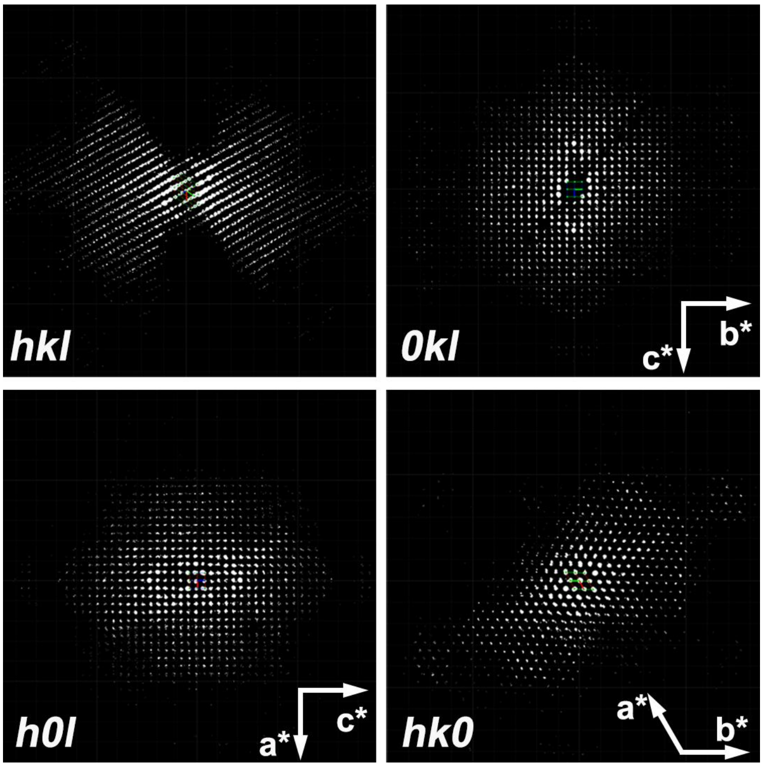

Supplementary Fig. 22. The projections of the 3D ED data of single-crystal GZU-3.

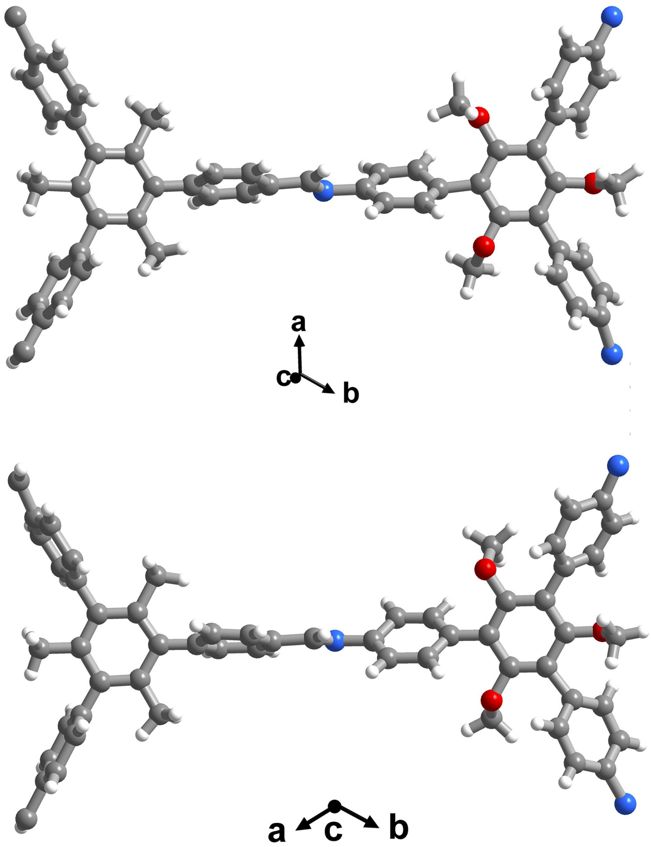

Supplementary Fig. 23. The dihedral angles of TOAB and TTFB between the middle and surrounding benzene segments shown in the asymmetric unit of single-crystal GZU-1, which is divided into two parts for clarity.

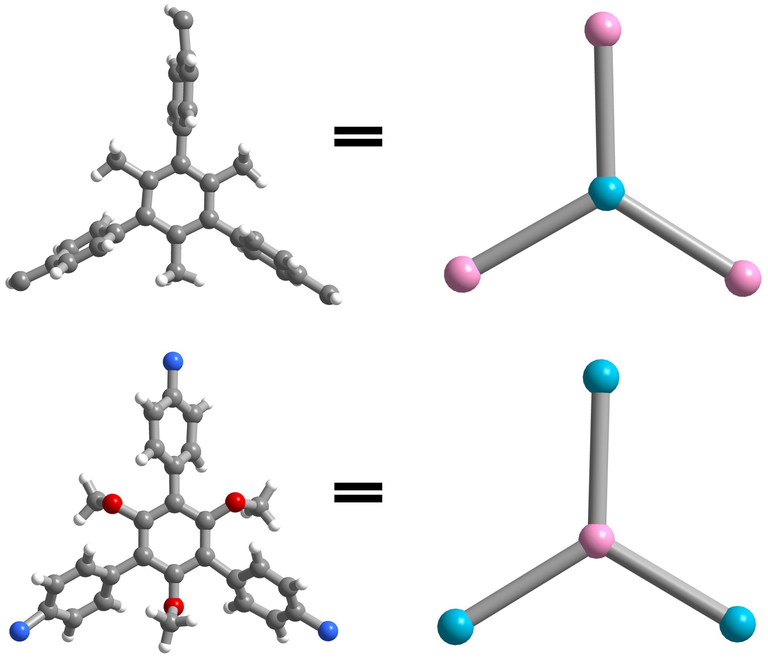

Supplementary Fig. 24. The TTFB and TOAB part in GZU-1 (simplified as three-connection nodes).

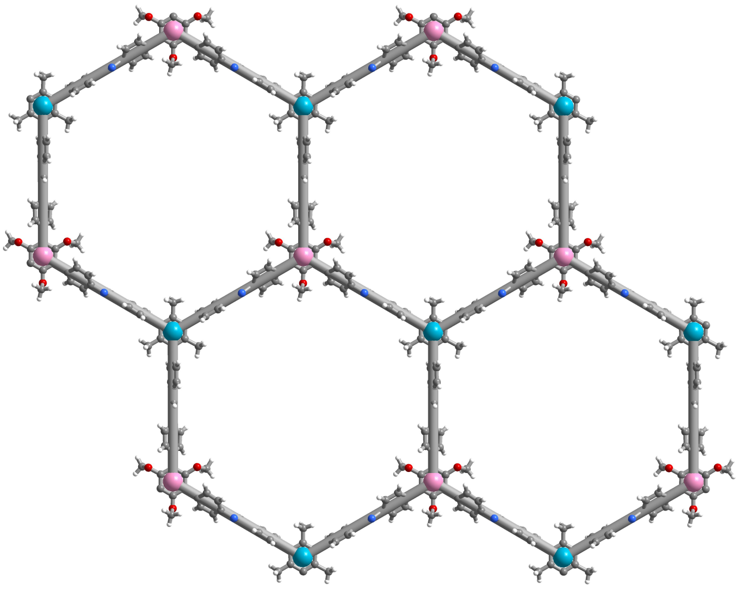

Supplementary Fig. 25. The single **hcb** network of GZU-1.

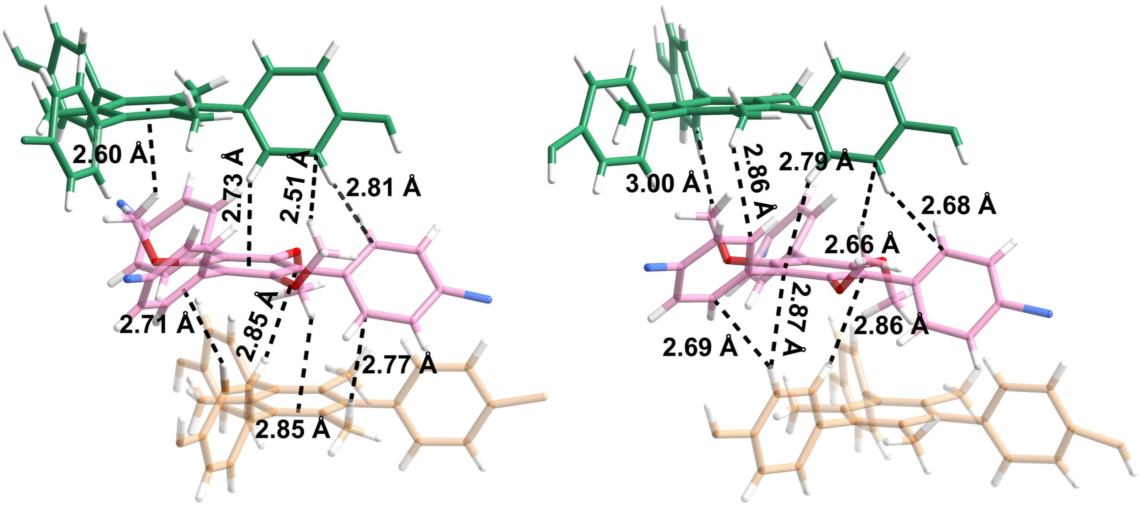

Supplementary Fig. 26. The interactions between the adjacent TTFB and TOAB in single-crystal GZU-1.

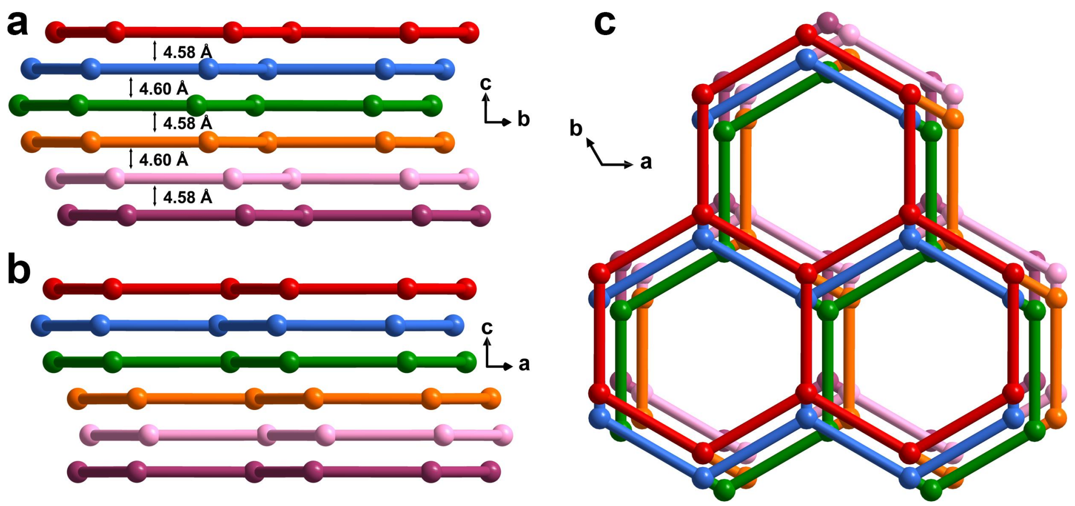

Supplementary Fig. 27. The view of topology packing structure of GZU-1 along a, b, and, c axis, respectively.

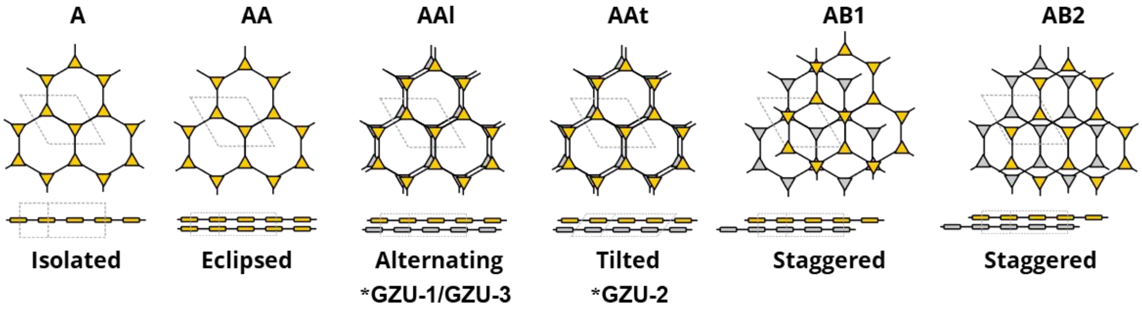

Supplementary Fig. 28. Different possible stacking patterns for 2D COFs based on a **hcb** network. For the canonical AA*l* and AA*t* stacking only two sheets are considered on the unit cell, AAl_2 and AAt_2, while GZU-1 and GZU-3 are better described as 6-fold alternating AA stacking (AAl_6) and GZU-2 as 4-fold shifted AA stacking (AAt_4).

The relative energy per COF sheet for all evaluated stacking patterns is presented on Supplementary Table 4. The results show that for GZU-1 (TTFB + TOAB) the experimental structure is the most stable. For GZU-2 and GZU-3 (TTAB + TOFB), the AAt_4 stacking of GZU-2 is slightly more stable than the AAl_6, nevertheless both structures are considerably more stable than all other possible stacking patterns.

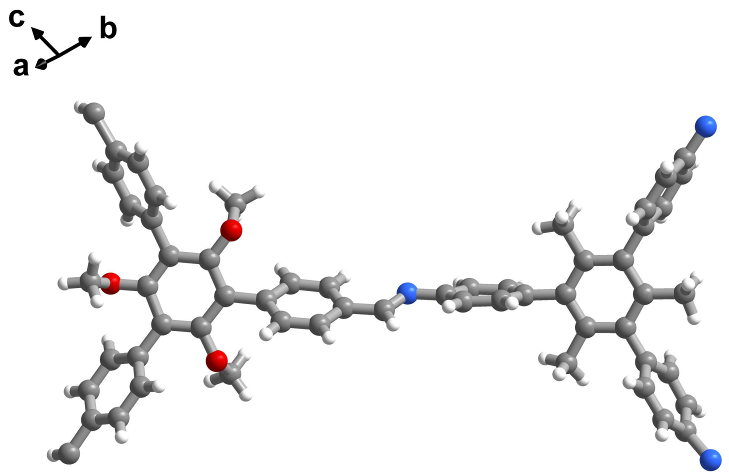

Supplementary Fig. 29. The dihedral angles of TTAB and TOFB between the middle and surrounding benzene segments shown in the asymmetric unit of single-crystal GZU-2.

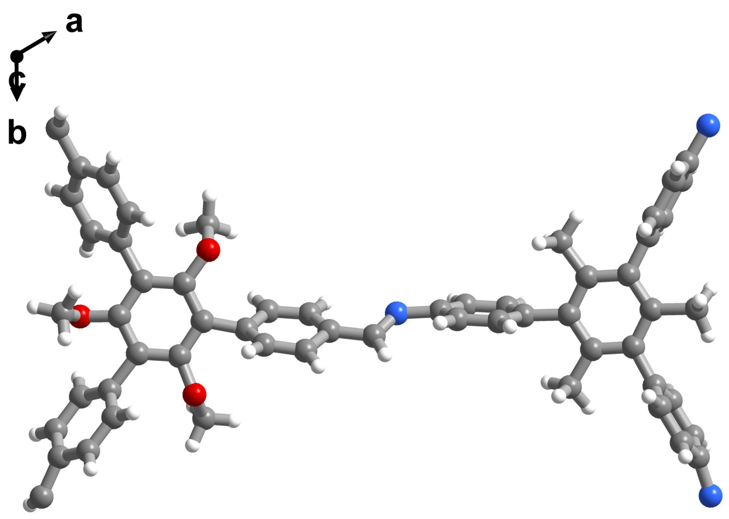

Supplementary Fig. 30. The dihedral angles of TTAB and TOFB between the middle and surrounding benzene segments shown in the asymmetric unit of single-crystal GZU-3.

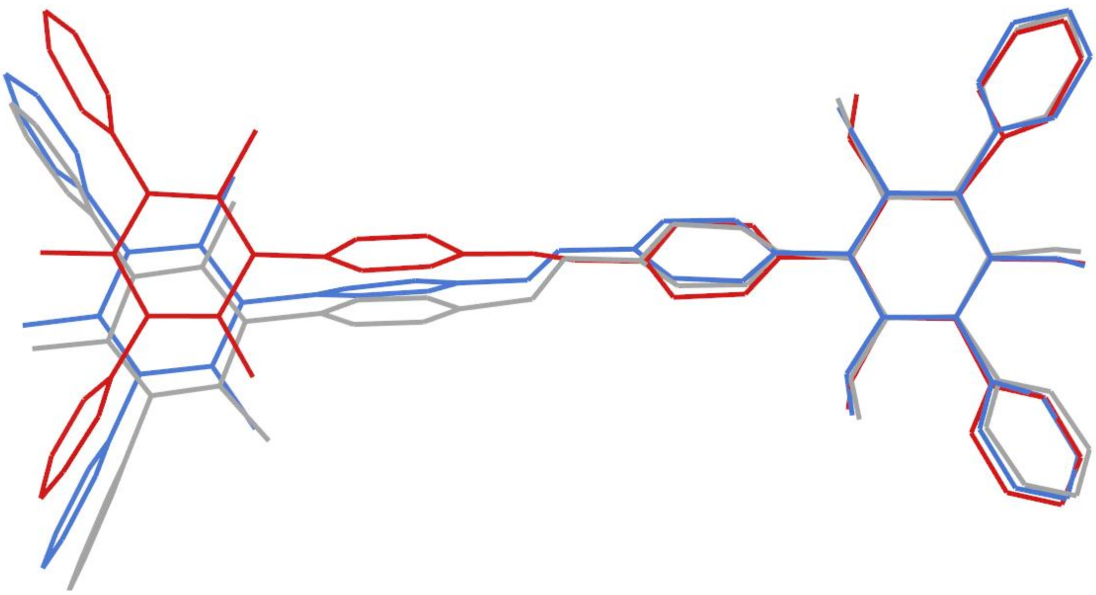

Supplementary Fig. 31. The superposition diagram of the asymmetric unit of GZU-1 (red), GZU-2 (blue), and GZU-3 (grey).

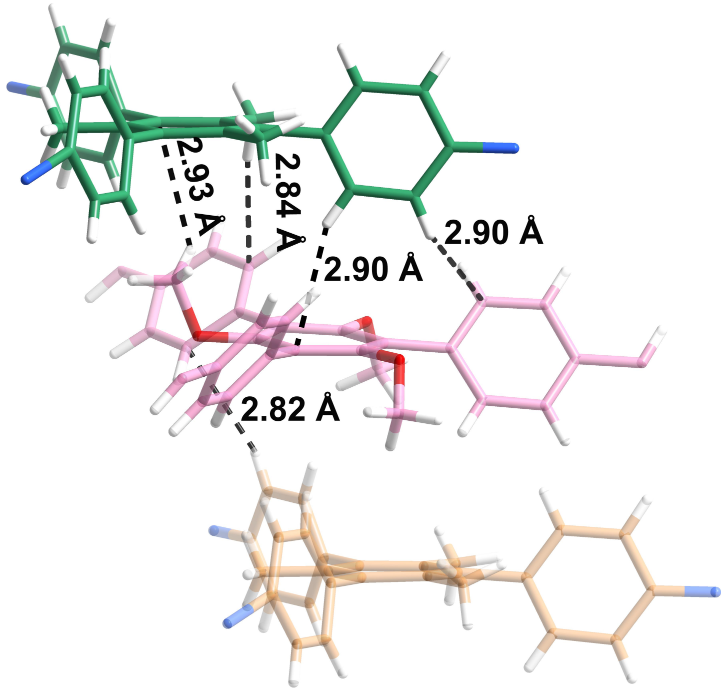

Supplementary Fig. 32. The interactions between the adjacent TTAB and TOFB in single-crystal GZU-2.

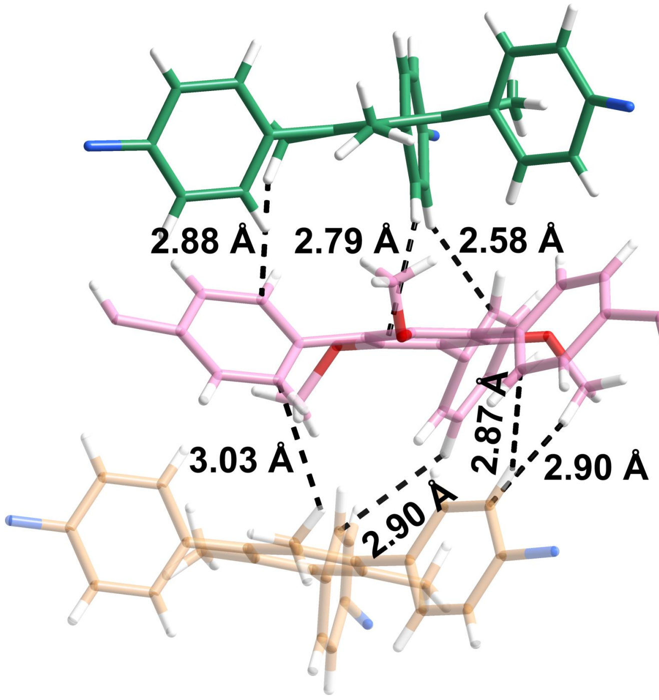

Supplementary Fig. 33. The interactions between the adjacent TTAB and TOFB in single-crystal GZU-3.

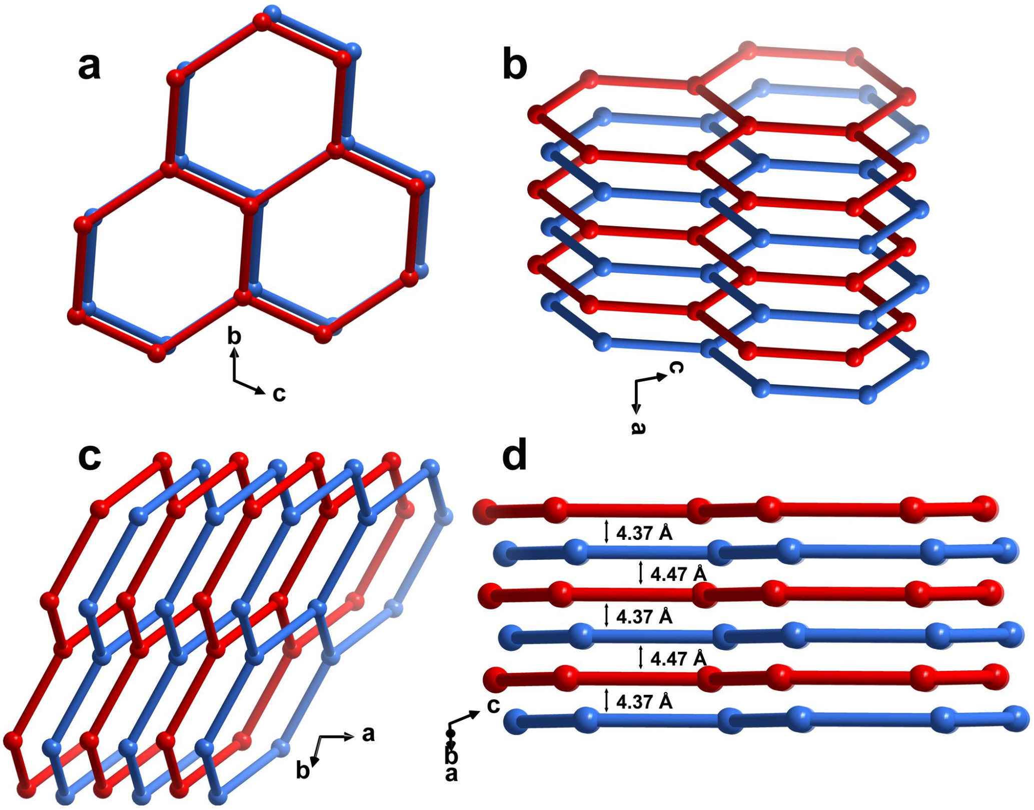

Supplementary Fig. 34. (a–c) The view of topology packing structure of GZU-2 along a, b, and, c axis, respectively. (d) The side view of topology packing structure of GZU-2.

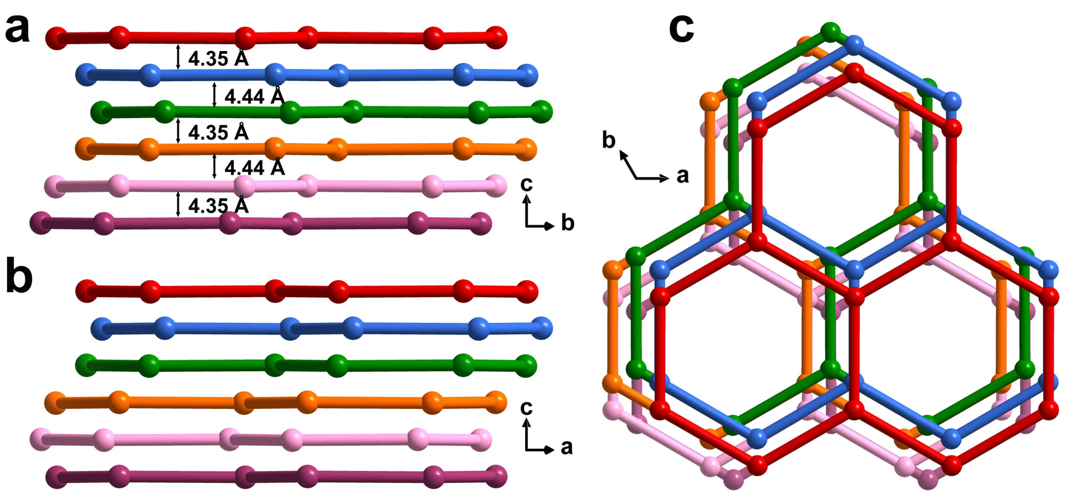

Supplementary Fig. 35. The view of topology packing structure of GZU-3 along a, b, and, c axis, respectively.

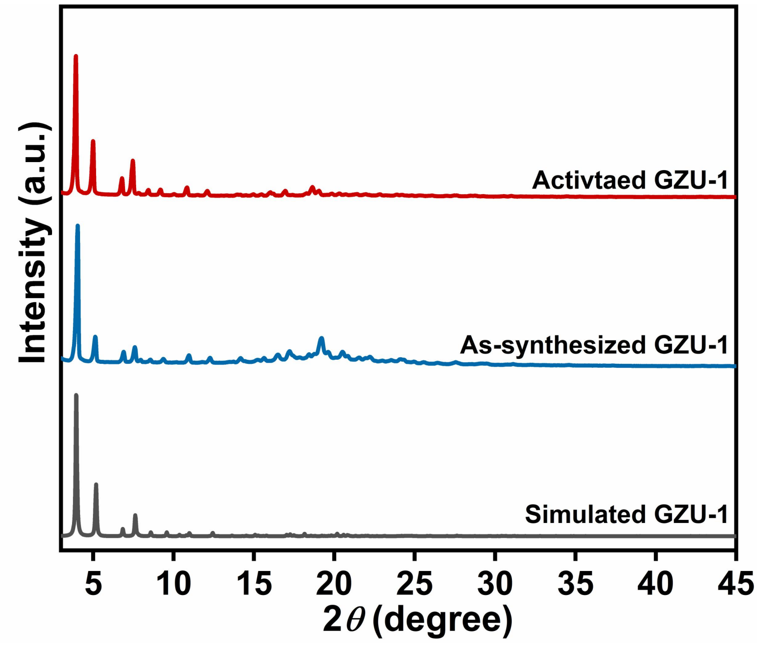

Supplementary Fig. 36. The PXRD patterns of as-synthesized and activated GZU-1 in comparison with the simulated curve according to the GZU-1 crystal structure derived from the 3D ED (the difference should be due to the solvent effect).

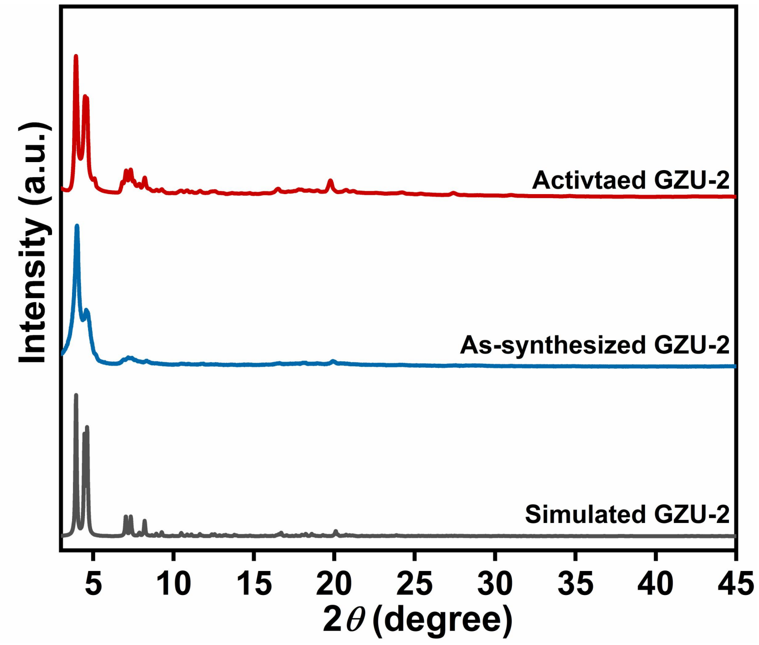

Supplementary Fig. 37. The PXRD patterns of as-synthesized and activated GZU-2 in comparison with the simulated curve according to the GZU-2 crystal structure derived from the 3D ED (the difference should be due to the solvent effect).

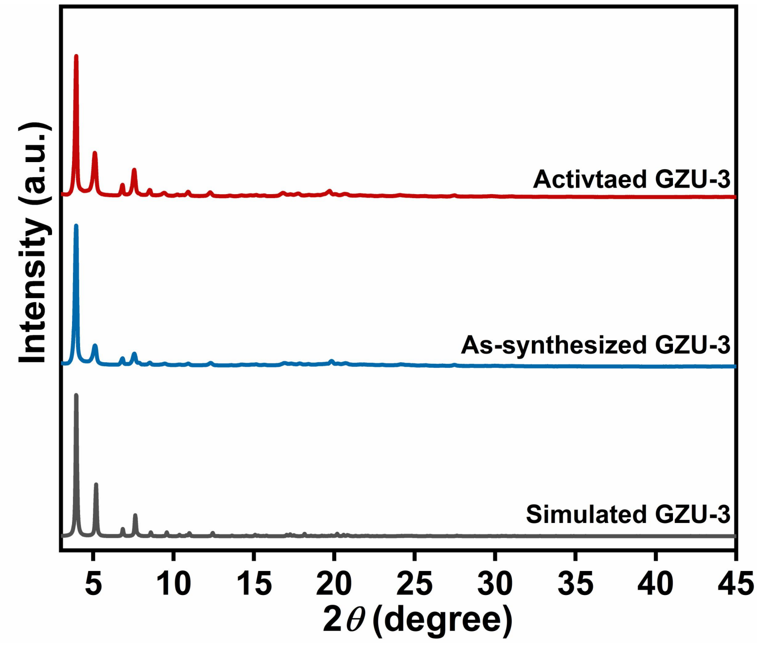

Supplementary Fig. 38. The PXRD patterns of as-synthesized and activated GZU-3 in comparison with the simulated curve according to the GZU-3 crystal structure derived from the 3D ED (the difference should be due to the solvent effect).

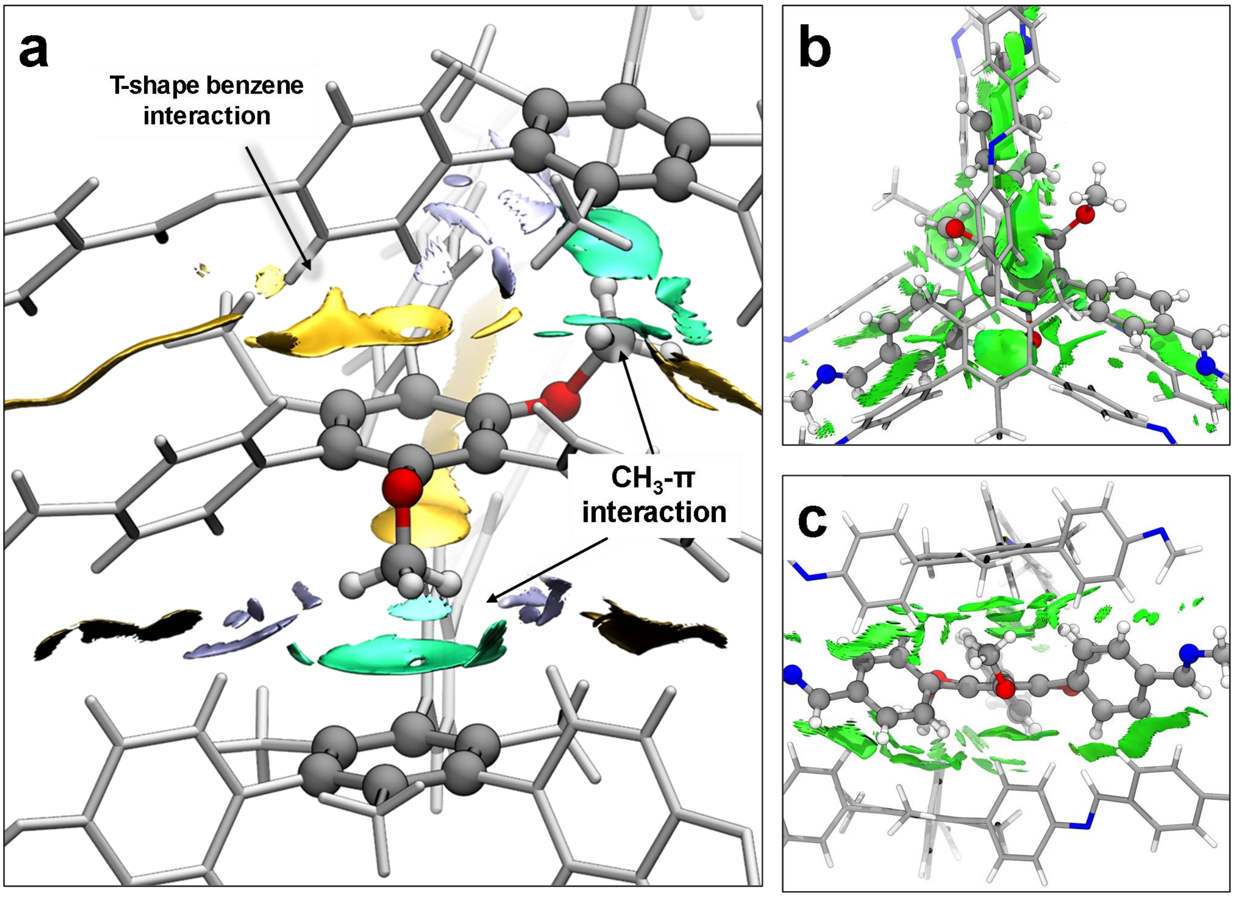

Supplementary Fig. 39. Non-covalent interactions (NCI) surface plots of the between GZU-1 COF layers. (a) The main interactions between the layers arises from the CH_3-π from the OMe groups and benzene from adjacent layers, represented in green, and the T-shaped benzene-benzene interactions , represented in yellow. Other small lateral interactions between the adjacent layers are represented in blue. Perpendicular (b) and in-plane (c) views with all the interactions between the layers. gray: carbon, white: hydrogen, red: oxygen, and blue: nitrogen. The NCI surface plots calculations were carried out using the QTAIM as implemented in the CRITIC2 software, based on the DFT electronic density.[11]

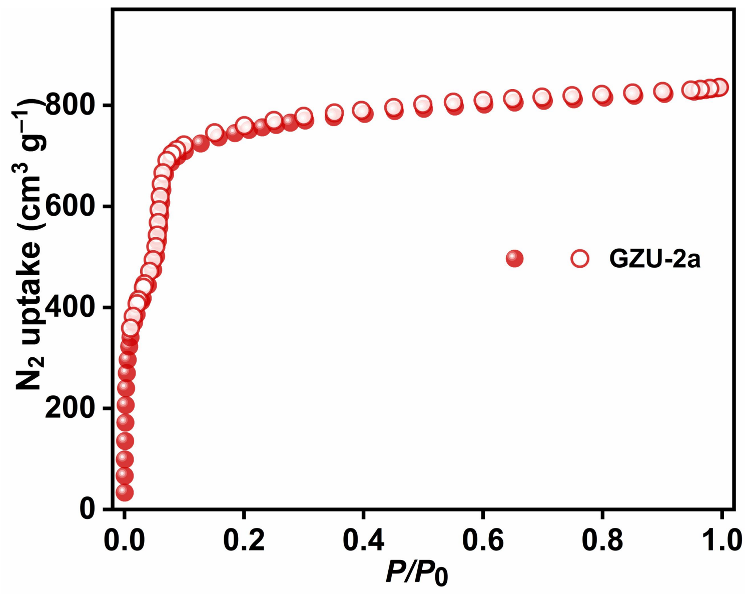

Supplementary Fig. 40. The N_2 adsorption (solid) and desorption (hollow) isotherms of GZU-2a at 77 K.

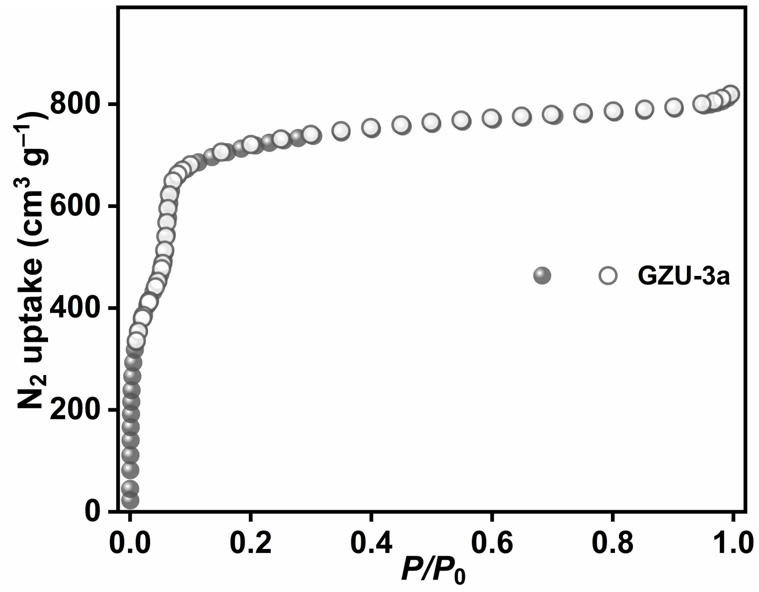

Supplementary Fig. 41. The N_2 adsorption (solid) and desorption (hollow) isotherms of GZU-3a at 77 K.

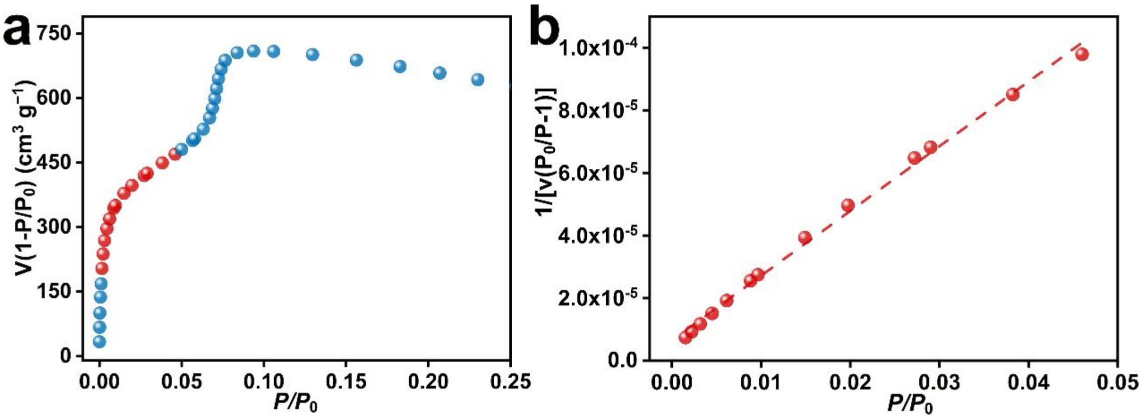

Supplementary Fig. 42. The BET surface area calculation for GZU-1a based on the N_2 adsorption isotherm at 77 K. (a) Rouquerol plot (red dot was selected for BET surface area calculation). (b) BET plot taken from $P/P_0 = 0.0015 – 0.046$. [C: the BET constant; V_m: the monolayer capacity]

The following values obtained for the BET Surface Area, Slope, Y-Intercept, C, V_m, R^2, $1/(\sqrt{C} + 1)$, and P/P_0 at V_m Using the Fittings.

**BET surface area**	2099.8700 ± 39.3132 $m^2\ g^{-1}$
**Slope**	0.002066 ± 0.000039 g cm^{-3}
***Y*-intercept**	0.000007 ± 0.000001 g cm^{-3}
C	317.785372
V_m	482.4428 $cm^3\ g^{-1}$
R^2	0.998
**1/(√C + 1)**	0.0531
P/P_0 **at** V_m	0.0432

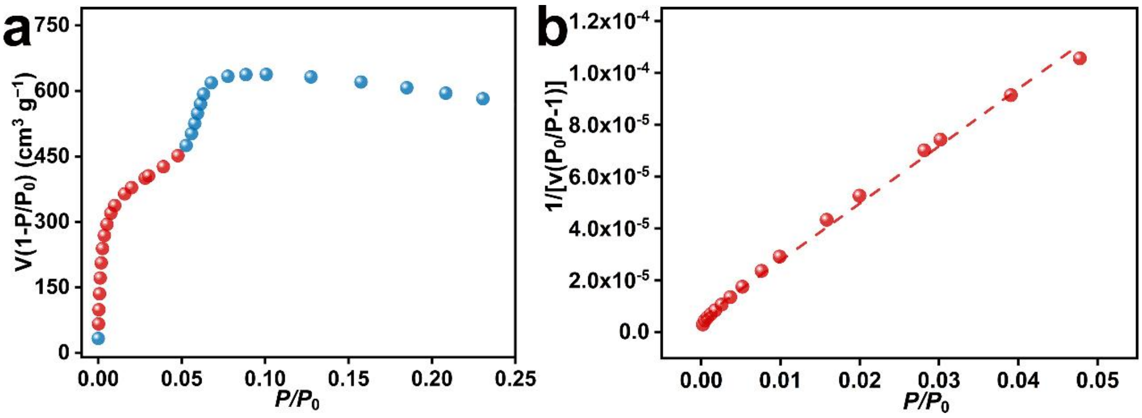

Supplementary Fig. 43. The BET surface area calculation for GZU-1a based on the N_2 adsorption isotherm at 77 K. (a) Rouquerol plot (red dot was selected for BET surface area calculation). (b) BET plot taken from $P/P_0 = 0.0002 – 0.048$.

The following values obtained for the BET Surface Area, Slope, *Y*-Intercept, *C*, V_m, R^2, 1/(√C + 1), and P/P_0 at V_m Using the Fittings. [*C*: the BET constant; V_m: the monolayer capacity]

**BET surface area**	1965.0422 ± 36.1635 $m^2\ g^{-1}$
**Slope**	0.002209 ± 0.000041 $g\ cm^{-3}$
***Y*-intercept**	0.000006 ± 0.000001 $g\ cm^{-3}$
C	400.628167
V_m	482.4428 $cm^3\ g^{-1}$
R^2	0.998
**1/(√C + 1)**	0.0476
**P/P_0 at nm**	0.0411

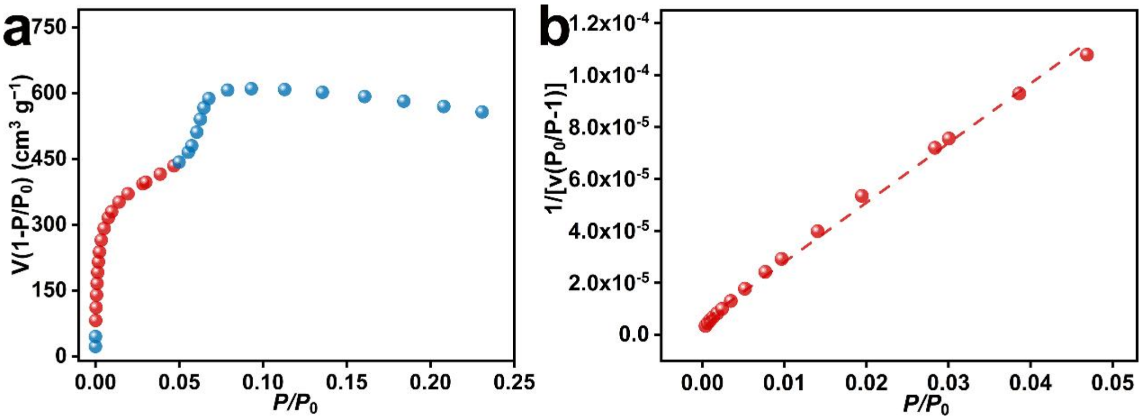

Supplementary Fig. 44. The BET surface area calculation for GZU-1a based on the N_2 adsorption isotherm at 77 K. (a) Rouquerol plot (red dot was selected for BET surface area calculation). (b) BET plot taken from $P/P_0 = 0.0003 – 0.048$.

The following values obtained for the BET Surface Area, Slope, *Y*-Intercept, *C*, V_m, R^2, $1/(\sqrt{C} + 1)$, and P/P_0 at V_m Using the Fittings. [*C*: the BET constant; V_m: the monolayer capacity]

BET surface area	1897.1534 ± 30.8696 m^2 g^{-1}
Slope	0.002289 ± 0.000037 g cm^{-3}
Y-intercept	0.000005 ± 0.000001 g cm^{-3}
C	450.471415
V_m	435.8689 cm^3 g^{-1}
R^2	0.998
$1/(\sqrt{C} + 1)$	0.0450
P/P_0 at nm	0.0397

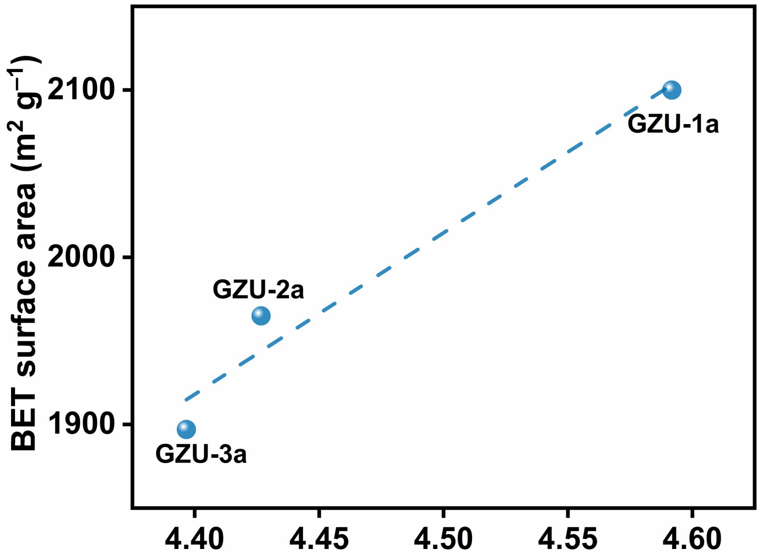

Supplementary Fig. 45. The relationship between the BET surface areas of GZU-1a, GZU-2a, and GZU-3a with the average interlayered distance.

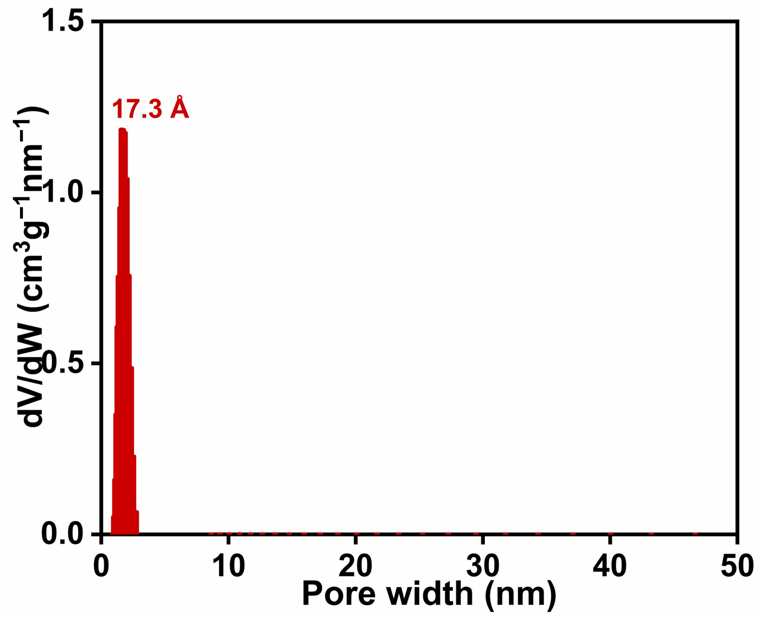

Supplementary Fig. 46. The pore size distribution of GZU-1a.

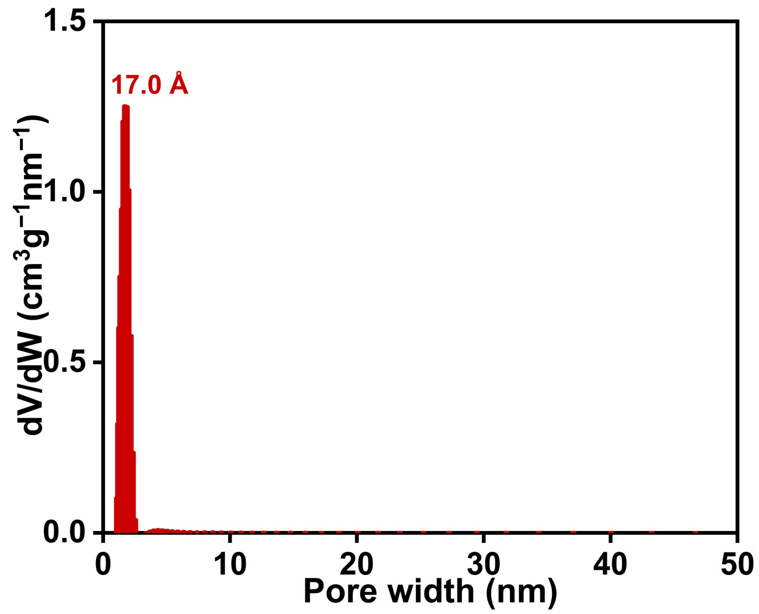

Supplementary Fig. 47. The pore size distribution of GZU-2a.

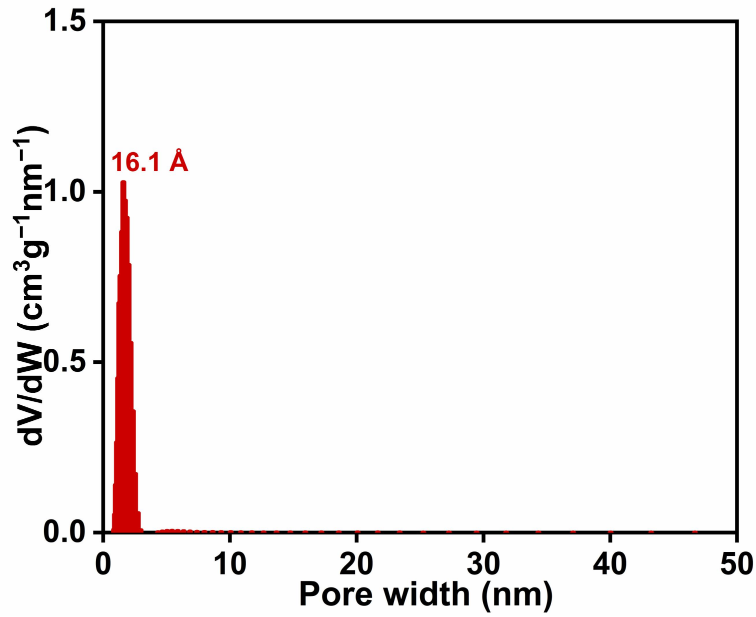

Supplementary Fig. 48. The pore size distribution of GZU-3a.

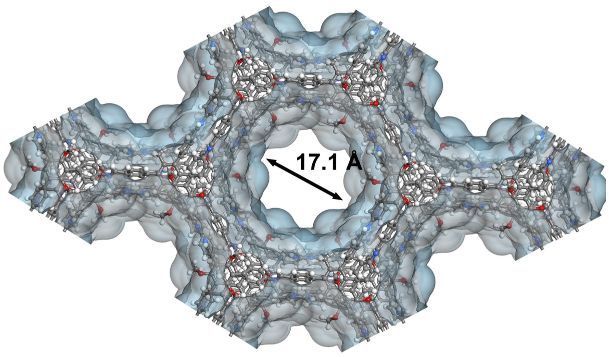

Supplementary Fig. 49. The stacking open channel of single-crystal GZU-1.

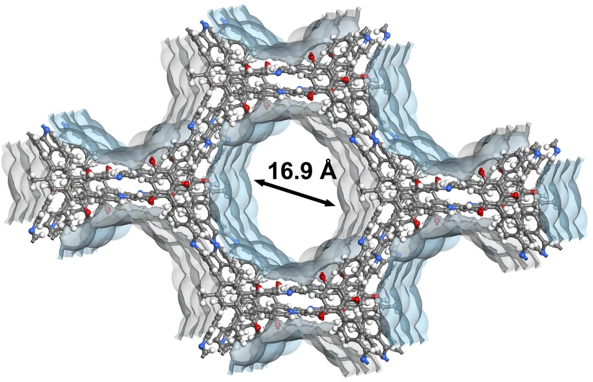

Supplementary Fig. 50. The stacking open channel of single-crystal GZU-2.

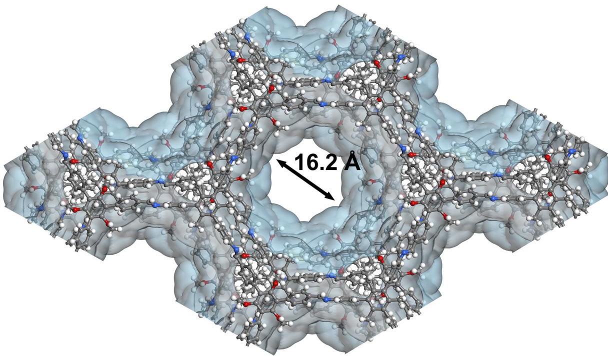

Supplementary Fig. 51. The stacking open channel of single-crystal GZU-3.

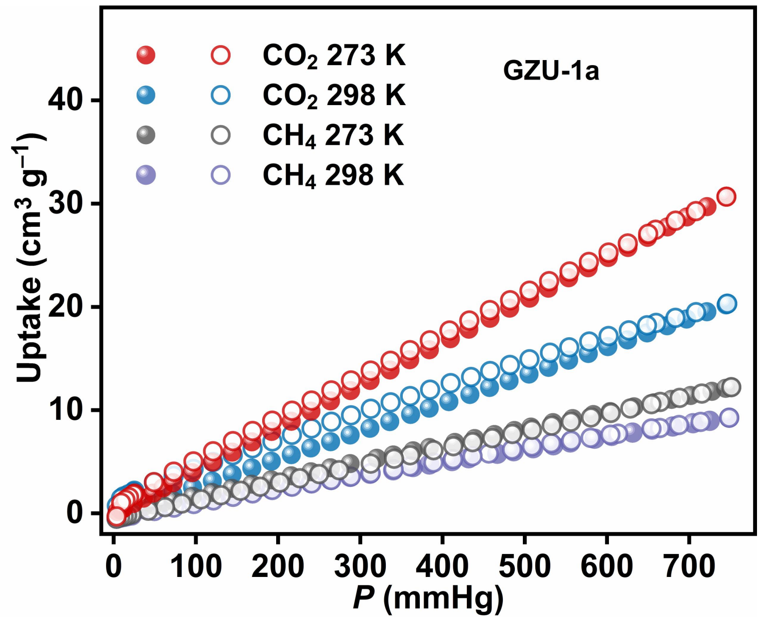

Supplementary Fig. 52. The CO_2 and CH_4 sorption isotherms of activated GZU-1 at 273 and 298 K.

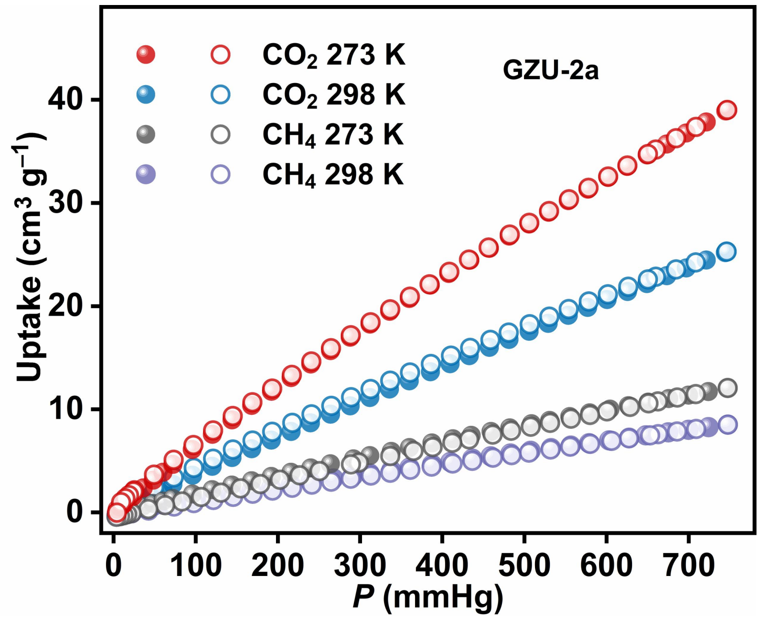

Supplementary Fig. 53. The CO_2 and CH_4 sorption isotherms of activated GZU-2 at 273 and 298 K.

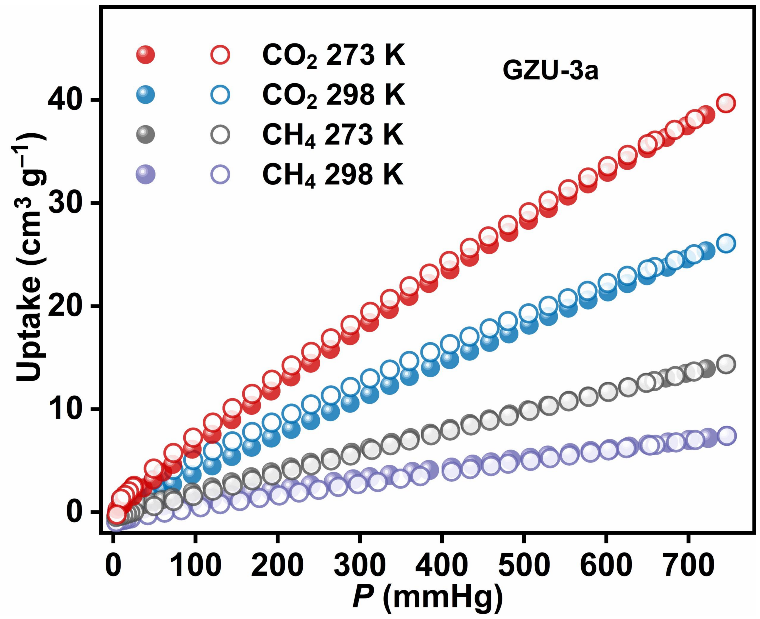

Supplementary Fig. 54. The CO_2 and CH_4 sorption isotherms of activated GZU-3 at 273 and 298 K.

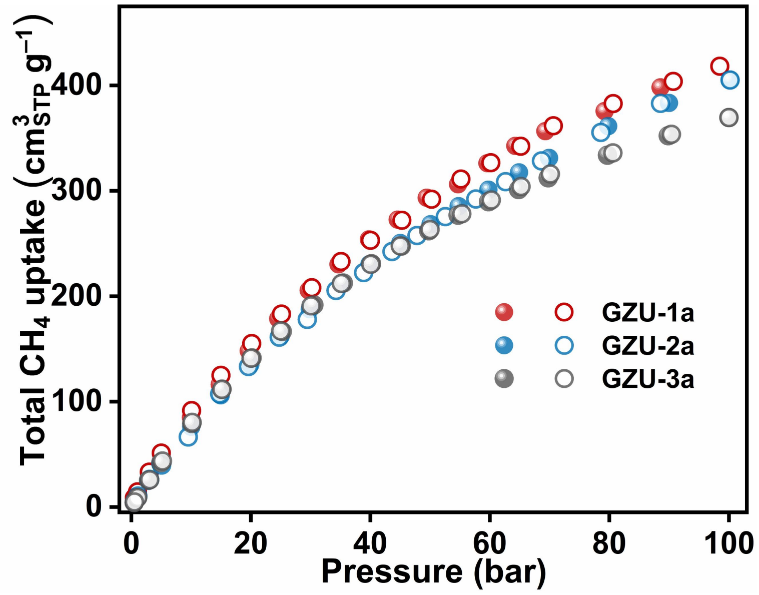

Supplementary Fig. 55. The high-pressure total methane uptake of GZU-1a, GZU-2a, and GZU-3a at 298 K.

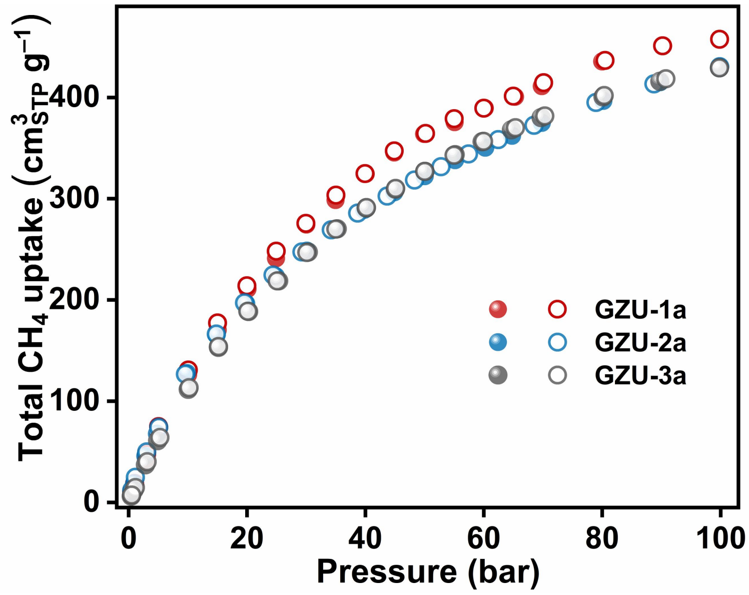

Supplementary Fig. 56. The high-pressure total methane uptake of GZU-1a, GZU-2a, and GZU-3a at 273 K.

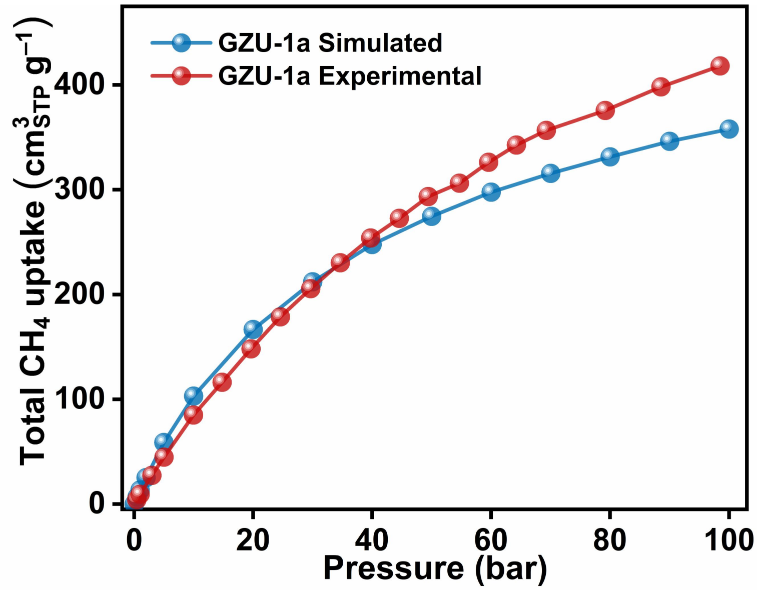

Supplementary Fig. 57. The comparison between experimental and simulated methane isotherms of GZU-1a at 298K.

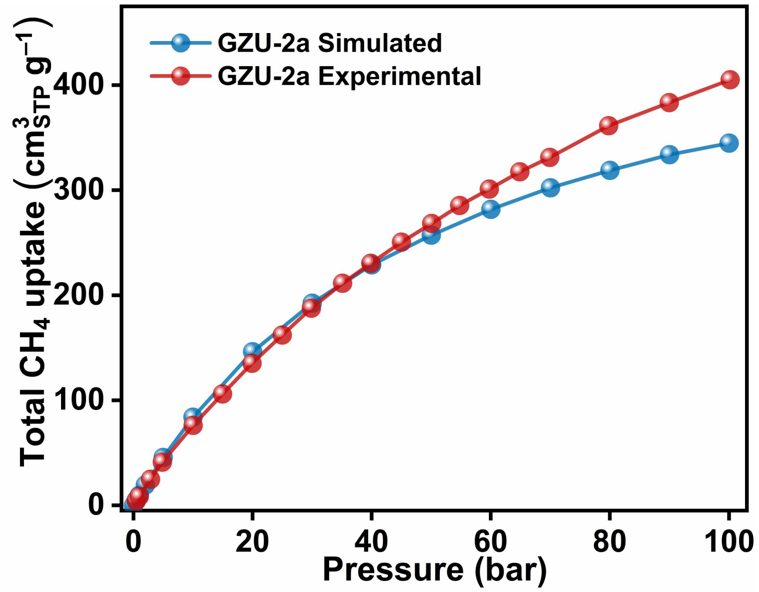

Supplementary Fig. 58. The comparison between experimental and simulated methane isotherms of GZU-2a at 298K.

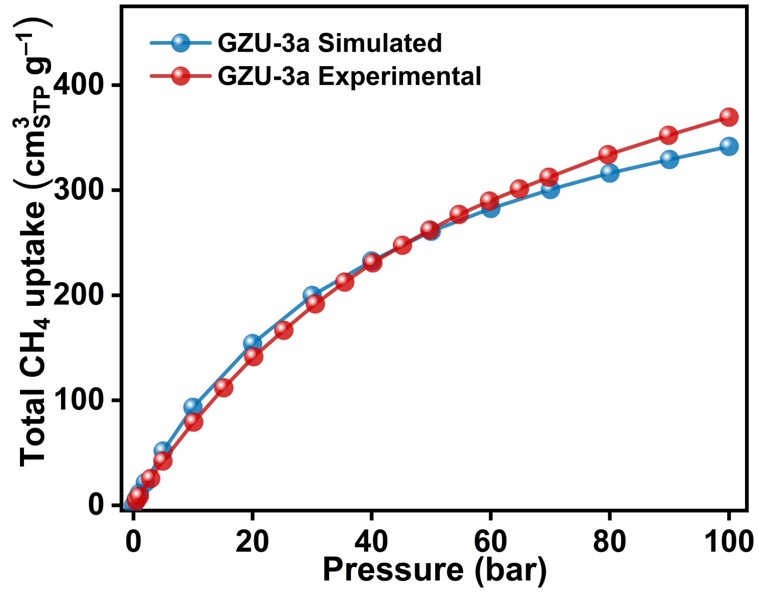

Supplementary Fig. 59. The comparison between experimental and simulated methane isotherms of GZU-3a at 298K.

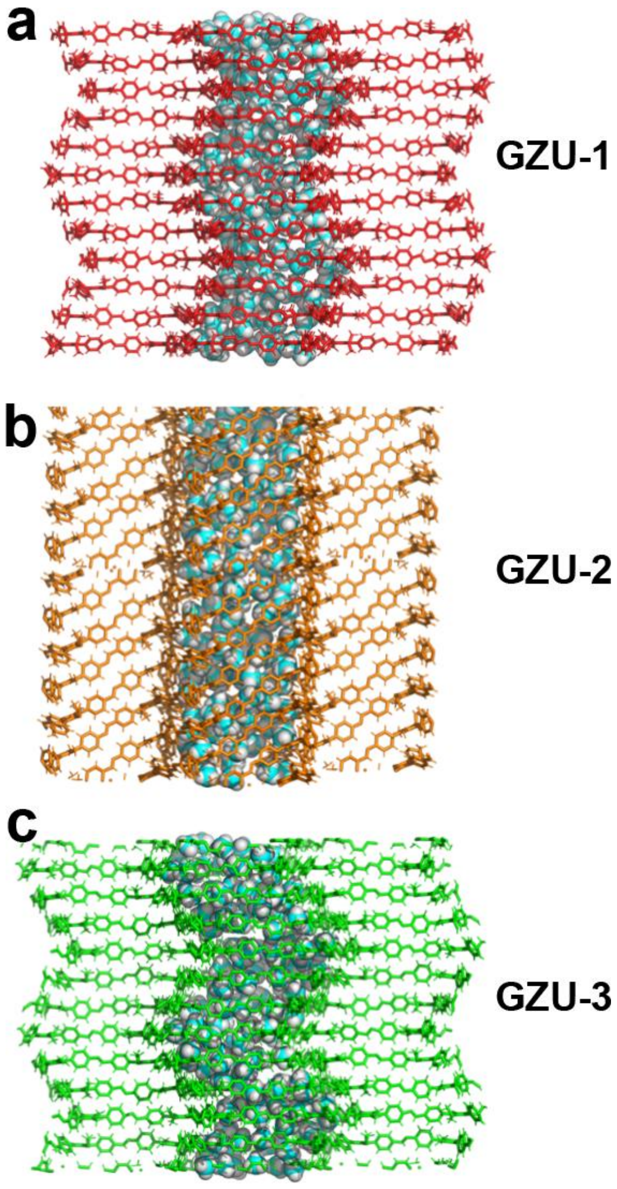

Supplementary Fig. 60. The snapshots of the GCMC simulations at 50 bar for a) GZU-1, b) GZU-2, c) GZU-3. The slipping of the COF sheets creates cavities and increase the surface area, favoring the large methane uptake. For clarity, only the molecules on the central pore are being shown.

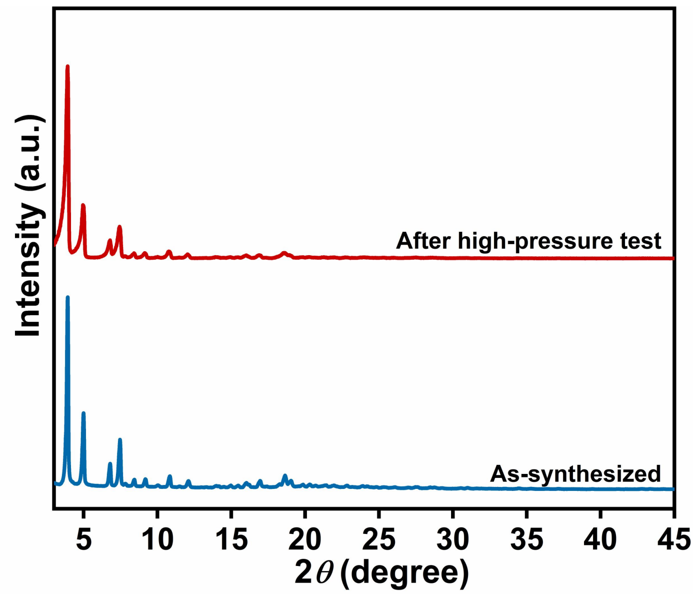

Supplementary Fig. 61. The PXRD patterns of GZU-1 after high pressure methane sorption experiment in comparison with the as-synthesized GZU-1.

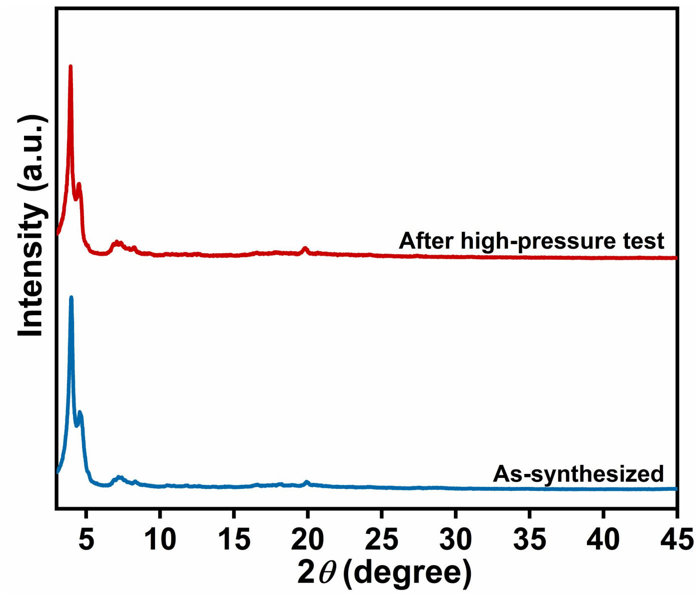

Supplementary Fig. 62. The PXRD patterns of GZU-2 after high pressure methane sorption experiment in comparison with the as-synthesized GZU-2.

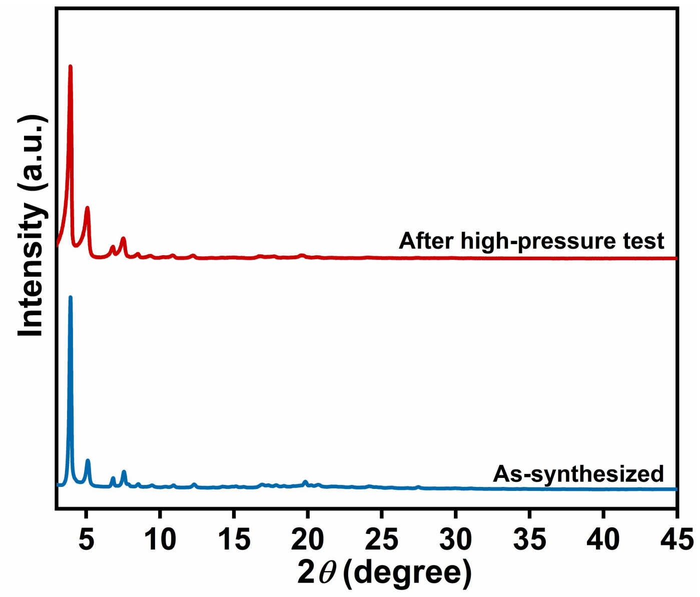

Supplementary Fig. 63. The PXRD patterns of GZU-3 after high pressure methane sorption experiment in comparison with the as-synthesized GZU-3.

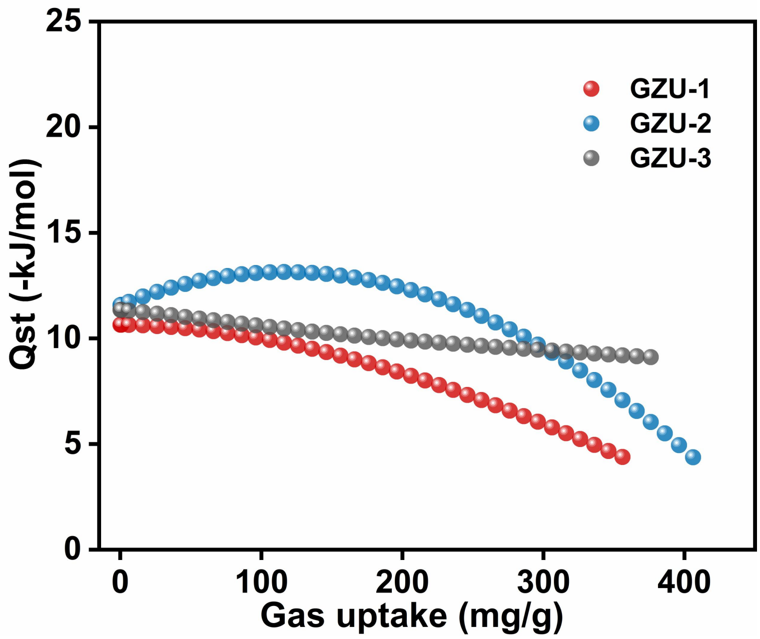

Supplementary Fig. 64. The coverage-dependent isosteric heat of methane for activated GZU-1, GZU-2, and GZU-3.

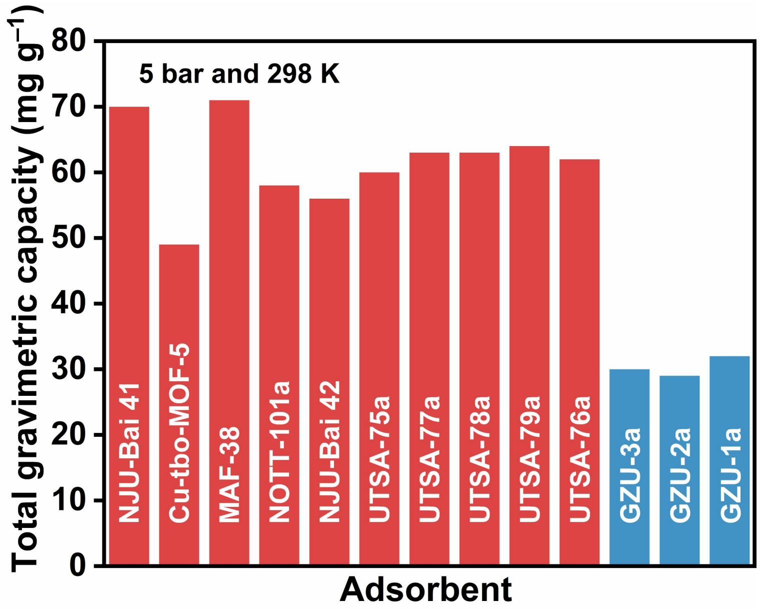

Supplementary Fig. 65. The total CH_4 gravimetric capacities of activated GZU-1, GZU-2, GZU-3 in comparison to other MOFs at 5 bar and 298 K.

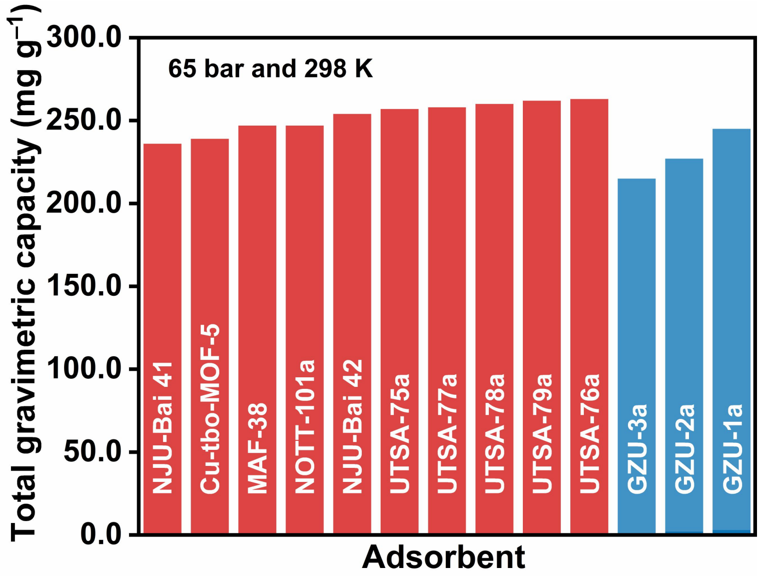

Supplementary Fig. 66. The total CH_4 gravimetric capacities of activated GZU-1, GZU-2, GZU-3 in comparison to other MOFs at 65 bar and 298 K.

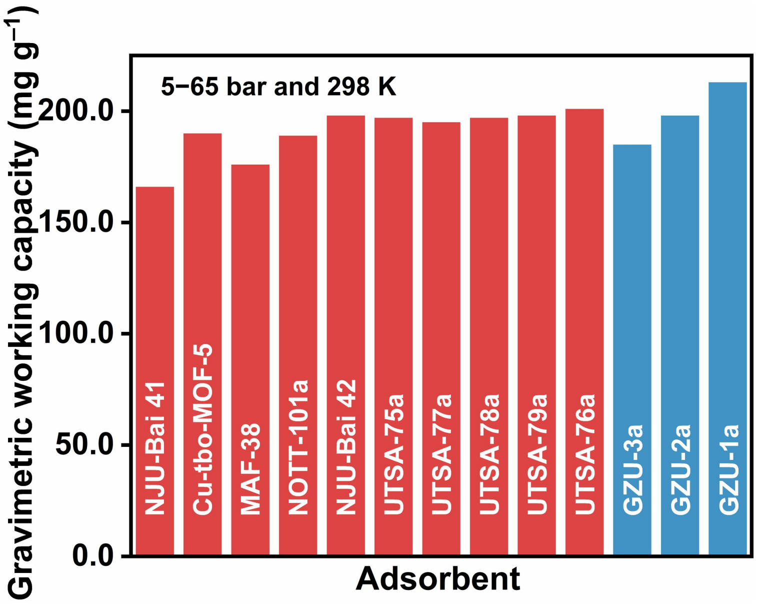

Supplementary Fig. 67. The CH_4 gravimetric working capacities of activated GZU-1, GZU-2, GZU-3 in comparison to other MOFs (5 to 65 bar and 298 K).

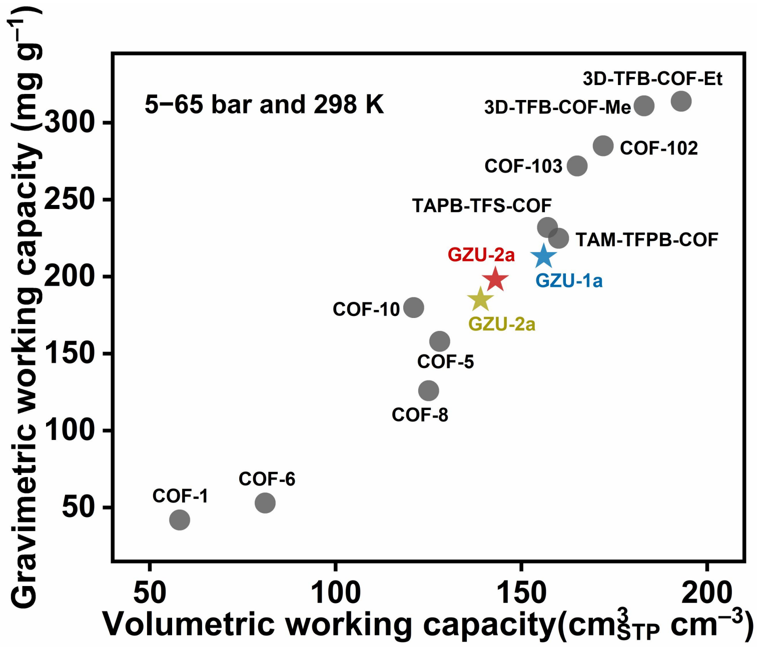

Supplementary Fig. 68. The methane gravimetric and volumetric working capacities of GZU-1a, GZU-2a, GZU-3a in comparison to other COFs reported to date.

Supplementary Table 1. The data collection and structure determination details of GZU-1.

**Compound**	**GZU-1**
CCDC number	2455725
Empirical formula	$C_{114}H_{89}N_6O_6$
Formula weight	1639.00
Temperature [K]	77.00
Crystal system	trigonal
Space group (number)	$P3_2$(145)
a [Å]	25.600(4)
b [Å]	25.600(4)
c [Å]	27.550(6)
α [°]	90
β [°]	90
γ [°]	120
Volume [Å^3]	15636(6)
Z	3
ρ_{calc} [gcm^{-3}]	0.522
μ [mm^{-1}]	0.000
F(000)	1074
Crystal colour	white
Crystal shape	block
Radiation	Electron (λ=0.02508 Å)
2Θ range [°]	0.08 to 1.35 (1.06 Å)
Index ranges	$-24 \leq h \leq 24$<br>$-23 \leq k \leq 23$<br>$-25 \leq l \leq 25$
Reflections collected	39589
Independent reflections	16682<br>R_{int} = 0.2614<br>R_{sigma} = 0.3037
Completeness	94.9 %
Data / Restraints / Parameters	16682/18/433
Goodness-of-fit on F^2	0.933
Final R indexes [$I \geq 2\sigma(I)$]	R_1 = 0.1127<br>wR_2 = 0.2676
Final R indexes [all data]	R_1 = 0.2311<br>wR_2 = 0.3483
Largest peak/hole [eÅ^{-3}]	0.27/-0.15

Supplementary Table 2. The RED data collection and structure determination details of GZU-2.

**Compound**	**GZU-2**
CCDC number	2415331
Empirical formula	$C_{57}H_{45}N_3O_3$
Formula weight	820.01
Temperature [K]	293(2)
Crystal system	triclinic
Space group (number)	$P\bar{1}$ (2)
a [Å]	10.855(2)
b [Å]	22.019(4)
c [Å]	24.556(5)
α [°]	111.76(3)
β [°]	95.02(3)
γ [°]	101.66(3)
Volume [Å^3]	5253(2)
Z	2
ρ_{calc} [gcm^{-3}]	0.518
μ [mm^{-1}]	0.000
F(000)	359
Crystal colour	white
Crystal shape	block
Radiation	Electron (λ=0.02508 Å)
2Θ range [°]	0.11 to 1.45 (0.99 Å)
Index ranges	$-10 \leq h \leq 10$<br>$-22 \leq k \leq 22$<br>$-24 \leq l \leq 24$
Reflections collected	34068
Independent reflections	10291<br>R_{int} = 0.2827<br>R_{sigma} = 0.2804
Completeness	90.8 %
Data / Restraints / Parameters	10291/7/260
Goodness-of-fit on F^2	0.748
Final R indexes [$I \geq 2\sigma(I)$]	R_1 = 0.1327<br>wR_2 = 0.3079
Final R indexes [all data]	R_1 = 0.2117<br>wR_2 = 0.4040
Largest peak/hole [eÅ^{-3}]	0.27/-0.16
Extinction coefficient	73(9)

Supplementary Table 3. The RED data collection and structure determination details of GZU-3.

**Compound**	**GZU-3**
CCDC number	2415329
Empirical formula	$C_{57}H_{45}N_3O_3$
Formula weight	820.01
Temperature [K]	77.00
Crystal system	hexagonal
Space group (number)	$P6_5$ (170)
a [Å]	25.810(4)
b [Å]	25.810(4)
c [Å]	26.380(5)
α [°]	90
β [°]	90
γ [°]	120
Volume [Å^3]	15219(5)
Z	6
ρ_{calc} [gcm^{-3}]	0.537
μ [mm^{-1}]	0.000
F(000)	1051
Crystal colour	white
Crystal shape	block
Radiation	Electron (λ = 0.02508 Å)
2Θ range [°]	0.08 to 1.80 (0.80 Å)
Index ranges	$-31 \leq h \leq 32$<br>$-32 \leq k \leq 32$<br>$-32 \leq l \leq 32$
Reflections collected	88587
Independent reflections	20262<br>R_{int} = 0.2842<br>R_{sigma} = 0.2326
Completeness	100.0 %
Data / Restraints / Parameters	20262/8/170
Goodness-of-fit on F^2	0.948
Final R indexes [$I \geq 2\sigma(I)$]	R_1 = 0.1401<br>wR_2 = 0.3466
Final R indexes [all data]	R_1 = 0.2389<br>wR_2 = 0.4131
Largest peak/hole [eÅ^{-3}]	0.24/-0.13
Extinction coefficient	27(5)

Supplementary Table 4. Relative energy of different possible staking patterns for the GZU COFs. All values are in in kJ mol^{-1} per COF sheet.

Structure	A	AA	AA*l*	GZU-1/3	AAt	GZU-2	AB1	AB2
TTFB + TOAB	183.1	72.9	62.4	0.0	69.0	-	121.9	141.0
TTAB + TOFB	184.3	74.2	65.6	5.2	74.2	0.0	134.8	101.5

References

1 Sahoo, A. K., Yadav, C. & Moorthy, J. N. Copper complexes of thiazolo[5,4-d]thiazole-based porous polymers: Efficient catalytic synthesis of 2-arylquinolines and 2-arylbenzothiazoles. *Appl. Catal., A* **671**, 119557 (2024).

2 Wan, W., Sun, J., Su, J., Hovmoller, S. & Zou, X. Three-dimensional rotation electron diffraction: software RED for automated data collection and data processing. *J. Appl. Crystallogr.* 46, 1863-1873 (2013).

3 Kabsch, W. Integration, scaling, space-group assignment and post-refinement. *Acta. Crystallogr. D* **66**, 133-144 (2010).

4 SCALE3 ABSPACK–A Rigaku Oxford Diffraction program for absorption corrections, Rigaku Oxford Diffraction, (2017).

5 Sheldrick, G. M. SHELXL-2014; University of Göttingen, Göttingen, Germany, (2014).

6 Wang, S. *et al.* Single-crystal 2D covalent organic frameworks for plant biotechnology. *J. Am. Chem. Soc.* **145**, 12155-12163 (2023).

7 Li, J. *et al.* Polycrystalline covalent organic frameworks: multiscale structural insights from morphological diversity to dynamic lattice evolution. *J. Am. Chem. Soc.* **147**, 26955-26968 (2025).

8 Lyu, H., Diercks, C. S., Zhu, C. & Yaghi, O. M. Porous crystalline olefin-linked covalent organic frameworks. *J. Am. Chem. Soc. 141*, 6848-6852 (2019).

9 Pütz, A. M. *et al.* Total scattering reveals the hidden stacking disorder in a 2D covalent organic framework. *Chem. Sci.* **11**, 12647–12654 (2020).

10 Spitler, E. L. *et al.* A 2D covalent organic framework with 4.7-nm pores and insight into its interlayer stacking. *J. Am. Chem. Soc.* **133**, 19416–19421 (2011).

11 Otero-de-la-Roza A, Johnson E. R. & Luaña V. Critic2: A program for real-space analysis of quantum chemical interactions in solids. *Comput. Phys. Commun.* **185**, 1007–1018 (2014)
